# The Hidden Diversity of Diatrypaceous Fungi in China

**DOI:** 10.3389/fmicb.2021.646262

**Published:** 2021-05-31

**Authors:** Haiyan Zhu, Meng Pan, Nalin N. Wijayawardene, Ning Jiang, Rong Ma, Dongqin Dai, Chengming Tian, Xinlei Fan

**Affiliations:** ^1^The Key Laboratory for Silviculture and Conservation of Ministry of Education, Beijing Forestry University, Beijing, China; ^2^Center for Yunnan Plateau Biological Resources Protection and Utilization, College of Biological Resource and Food Engineering, Qujing Normal University, Yunnan, China; ^3^College of Forestry and Horticulture, Xinjiang Agricultural University, Ürümqi, China

**Keywords:** *Allocryptovalsa*, *Diatrype*, *Diatrypella*, *Eutypella*, fungal diversity, phylogeny, taxonomy

## Abstract

In this study, we investigated the diversity of diatrypaceous fungi from six regions in China based on morpho-molecular analyses of combined ITS and *tub2* gene regions. We accept 23 genera in *Diatrypaceae* with 18 genera involved in the phylogram, and the other five genera are lacking living materials with sequences data. Eleven species included in four genera (*viz*. *Allocryptovalsa*, *Diatrype*, *Diatrypella*, and *Eutypella*) have been isolated from seven host species, of which nine novel species (*viz*. *Allocryptovalsa castaneae*, *A. castaneicola*, *Diatrype betulae*, *D. castaneicola*, *D. quercicola*, *Diatrypella betulae*, *Da. betulicola*, *Da. hubeiensis*, and *Da. shennongensis*), a known species of *Diatrypella favacea*, and a new record of *Eutypella citricola* from the host genus *Morus* are included. Current results show the high diversity of *Diatrypaceae* which are wood-inhabiting fungi in China.

## Introduction

*Diatrypaceae* is an important family in *Xylariales* (*Sordariomycetes, Ascomycota*), containing many taxa with a worldwide distribution (Glawe and Jacobs, 1987; Mayorquin et al., 2016; Senwanna et al., 2017; Moyo et al., 2018a, b; Konta et al., 2020; Wijayawardene et al., 2020). Species of the *Diatrypaceae* are frequently saprobic on the decaying wood of angiosperms (Tendulkar, 1970; Acero et al., 2004; de Almeida et al., 2016). However, an endophyte of *Picea abies*, a gymnosperm host, was identified as an asexual morph of *Diatrypaceae* (*Libertella* sp.) by Caroll et al. (1977). Few endophytes such as *Diatrypella frostii* and *Peroneutypa scoparia* were reported later (de Errasti et al., 2010; Vieira et al., 2011). Strikingly, several plant pathogens of *Diatrypaceae* were reported causing canker, dieback, and grapevine trunk diseases, e.g., *Cryptosphaeria populina* was linked to canker in *Populus* species (Glawe and Rogers, 1984); *Cryptosphaeria pullmanensis* caused canker disease in *Populus alba* and *Salix alba* (Ma et al., 2016); *Cryptovalsa ampelina* caused grapevine trunk disease on *Vitis* species (Luque et al., 2006); *Eutypa lata* was isolated from *Prunus armeniaca* and *Vitis* species with canker and dieback symptoms (Lardner et al., 2005); *Eutypa leptoplaca* contributed to the dieback of grapevines (Trouillas and Gubler, 2004; Catal et al., 2007); and *Eutypella parasitica* caused canker in *Acer* species (Rappaz, 1987).

Nitschke (1867) proposed the first important study of *Diatrypaceae* (as *Diatrypeae*) including five genera (Supplementary Table 1). Kirk et al. (2001) accepted nine genera. Later, the family was considered to accommodate 13 genera by Kirk et al. (2008) (Supplementary Table 1). *Diatrypasimilis*, *Monosporascus*, and *Pedumispora* have been added to the family in subsequent studies (Abdel-Wahab et al., 2014; Klaysuban et al., 2014; Maharachchikumbura et al., 2015). Wijayawardene et al. (2018) reported 17 genera in the family (Supplementary Table 1). Subsequently, Dayarathne et al. (2016), Senwanna et al. (2017), Phookamsak et al. (2019) respectively introduced *Allocryptovalsa, Halodiatrype*, and *Neoeutypella*, and accepted them as members of *Diatrypaceae*. Wijayawardene et al. (2020) listed 20 genera in the family. Later, Dayarathne et al. (2020a, b), Konta et al. (2020) respectively added *Allodiatrype*, *Halocryptosphaeria*, and *Halocryptovalsa* to this family.

The sexual morph members of *Diatrypaceae* are characterized by perithecial ascomata usually with ostiolar necks, 8-spored or polysporous asci with a very long pedicel and J-/J+ apical apparatus, and allantoid ascospores (Senanayake et al., 2015; Dayarathne et al., 2016; de Almeida et al., 2016). Several asexual genera included in coelomycetes or hyphomycetes (*viz*. *Cytosporina*, *Libertella*, and *Phaeoisaria*) have been linked to the family *Diatrypaceae* (Dayarathne et al., 2016; de Almeida et al., 2016; Mehrabi et al., 2016; Shang et al., 2017). However, the asexual morphs of many species were still indistinguishable (Acero et al., 2004). Senanayake et al. (2015) summarized that the asexual morph of this family had acervular and astromatic conidiomata, branched conidiophores and filiform, allantoid or rarely straight conidia with flattened base and blunt apex. However, in most cases, it is difficult to differentiate diatrypaceous species based on asexual morphs (Glawe and Rogers, 1986; de Almeida et al., 2016).

Due to overlapping phenotypic characters in *Diatrypaceae*, polyphasic approaches to solve the taxonomy of fungi were very common in recent studies (Wijayawardene et al., 2016; Norphanphoun et al., 2017; Fan et al., 2018, 2020; Lawrence et al., 2018; Zhu et al., 2020). The first molecular phylogenetic analysis of *Diatrypaceae* based on ITS showed that *Cryptosphaeria*, *Diatrype*, *Diatrypella*, *Eutypa*, and *Eutypella* were polyphyletic (Acero et al., 2004). Recently, the identification and classification of diatrypaceous taxa were performed by the multiple sequence data (mostly ITS and *tub2*) and morphological characters (Phookamsak et al., 2019; Dayarathne et al., 2020a; Hyde et al., 2020b; Konta et al., 2020). Moreover, Konta et al. (2020) introduced one new genus *Allodiatrype* and five new species belonging to *Allocryptovalsa*, *Allodiatrype*, and *Diatrypella* from palms (*Arecaceae*) based on this criterion.

During the investigation of forest pathogens in China, 86 diatrypaceous specimens associated with various disease symptoms were collected from Beijing City, Xinjiang Uygur Autonomous Region, and four other provinces in China *viz*. Hubei, Hebei, Jiangsu, and Yunnan. The objectives were to supplement a multi-gene DNA dataset of *Diatrypaceae* including ITS and *tub2*, improve the phylogenetic systematics of this family, and provide a theoretical basis for the identification of diseases and pathogens.

## Methods

### Isolates

Symptomatic branches or twigs were collected from seven tree hosts (*Betula albosinensis*, *B. davurica*, *B. platyphylla*, *Castanea mollissima*, *Juglans regia*, *Morus alba*, and *Quercus mongolica*) from Beijing City, Xinjiang Uygur Autonomous Region, and four other provinces in China *viz*. Hubei, Hebei, Jiangsu, and Yunnan. Eighty-six fresh specimens of *Diatrypaceae* were put into envelopes with records of their altitude, collector, collecting time, host, longitude, and latitude. A total of 21 representative isolates were obtained by removing the ascospores or conidial mass from fresh specimens on the surface of 1.8% potato dextrose agar (PDA) and incubating at 25°C for 24 h. Single germinating spore was transferred onto a fresh PDA plate. Specimens and isolates were deposited in the Beijing Forestry University (BJFU) and the Beijing Museum of Natural History (BJM). Strains of the new species are maintained in the China Forestry Culture Collection Centre (CFCC).

### Morphological Analysis

Species identification was based on morphological features of fruiting bodies and micromorphology supplemented by cultural characteristics. Macro-morphological observations including structure and size of stromata, ectostromatic disc, and ostioles were determined using a Leica stereomicroscope (M205 FA) (Leica Microsystems, Wetzlar, Germany). Micro-morphological photographs were captured using a Nikon Eclipse 80i microscope (Nikon Corporation, Tokyo, Japan), including conidiophores, asci, and conidia/ascospores. Adobe Bridge CS v. 6 and Adobe Photoshop CS v. 5 were used for manual editing. At least 10 conidiomata/ascomata, 10 asci, and 30 conidia/ascospores were randomly selected for measurement to calculate the mean width/length and respective standard deviations (SD). Cultural characteristics of strains incubated in the dark at 25°C were recorded. Colony morphology was described using the color charts of Rayner (1970). Nomenclatural novelties were deposited in the MycoBank (^1^ Crous et al., 2004).

### DNA Extraction, PCR Amplification, and Sequencing

Fungal mycelium grown on the cellophane on PDA was scraped for the extraction of genomic DNA following the modified CTAB method (Doyle and Doyle, 1990). Two loci were amplified, including the internal transcribed spacer (ITS) region and partial beta-tubulin (*tub2*) using the primer pairs ITS1/ITS4 (White et al., 1990) and T1/Bt2b (Glass and Donaldson, 1995; O’Donnell and Cigelnik, 1997), respectively. The additional combination of Bt2a and Bt2b (Glass and Donaldson, 1995) was used in case of amplification failure of the primer T1 and Bt2b. The polymerase chain reaction (PCR) assay was conducted as described in Fan et al. (2020). PCR amplification products were estimated via electrophoresis in 2% agarose gels. DNA sequencing was performed using an ABI PRISM^®^ 3730XL DNA Analyzer with a BigDye Terminater Kit v. 3.1 (Invitrogen, United States) at the Shanghai Invitrogen Biological Technology Company Limited (Beijing, China).

### DNA Sequence Analysis

The initial identities of our strains sequenced were obtained by morphological observations and nucleotide BLAST search. To clarify the phylogenetic position, the alignment based on a combined matrix using ITS and *tub2* sequences was performed to compare with other available species in *Diatrypaceae*. Reference sequences were selected based on ex-type or ex-epitype sequences available from relevant recently published literature (de Almeida et al., 2016; Senwanna et al., 2017; Shang et al., 2017, 2018; Hyde et al., 2019, 2020a; Phookamsak et al., 2019; Dayarathne et al., 2020a, b; Konta et al., 2020; Supplementary Table 2). *Xylaria hypoxylon* (CBS 122620) was selected as the outgroup. For each gene, sequences were aligned using MAFFT v. 7 (Katoh and Standley, 2013) and manually improved where necessary using MEGA v. 6 (Tamura et al., 2013). Ambiguously aligned sequences were excluded from the analysis. Alignments were used to infer a preliminary phylogenetic relationship for our sequences based on Maximum Parsimony (MP) with PAUP v. 4.0b10 (Swofford, 2003), Maximum Likelihood (ML) with PhyML v. 3.0 (Guindon et al., 2010), and Bayesian Inference (BI) analyses with MrBayes v. 3.1.2 (Ronquist and Huelsenbeck, 2003).

Maximum parsimony analysis was performed using a heuristic search option of 1,000 random-addition sequences. The tree bisection and reconnection (TBR) was selected as option to the branch swapping algorithm (Swofford, 2003). The branches of zero length were collapsed, and all equally parsimonious trees were saved. Clade stability was assessed with a bootstrap analysis of 1,000 replicates (Hillis and Bull, 1993). Tree length (TL), consistency index (CI), retention index (RI), and rescaled consistency (RC) were calculated (Swofford, 2003). ML analysis including 1,000 bootstrap replicates (Hillis and Bull, 1993) was conducted with a general time reversible (GTR) model of site substitution, including gamma-distributed rate heterogeneity and a proportion of invariant sites (Guindon et al., 2010). The nucleotide model of evolution for each of the data partitions were estimated by MrModeltest v. 2.3 (Posada and Crandall, 1998) before the Bayesian analysis. BI analysis was performed using a Markov Chain Monte Carlo (MCMC) algorithm with Bayesian posterior probabilities (Rannala and Yang, 1996). Two MCMC chains were run for 1,000,000 generations with a sampling frequency at every 100th generation. The first 25% of trees were discarded as the burn-in phase of each analysis, and the posterior probabilities (BPP) were calculated to assess the remaining trees (Rannala and Yang, 1996). The branch support from MP and ML analyses were evaluated with a bootstrapping (BS) method of 1,000 replicates (Hillis and Bull, 1993). The resulting trees were plotted in Figtree v. 1.4.4 and edited in Adobe Illustrator CS6 v. 16.0.0. All sequences from this study were deposited in GenBank (Supplementary Table 2). The multi-gene sequence alignment files were submitted to TreeBASE (^2^ accession number: S27126).

## Results

### Phylogenetic Analyses

**FIGURE 1:**
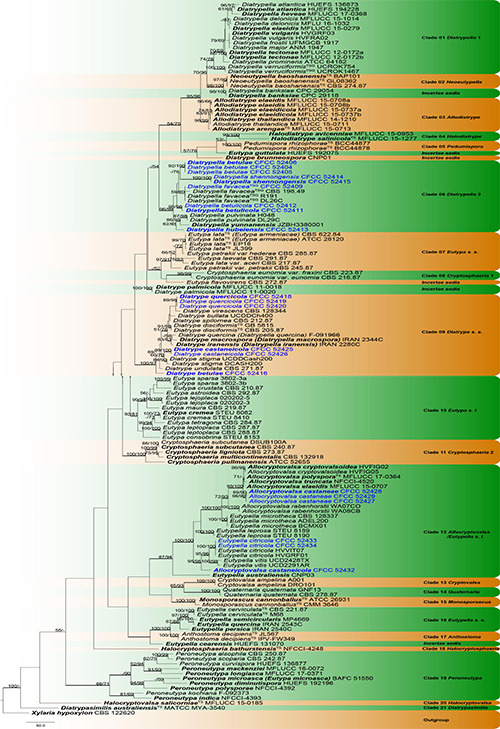
Phylogram of *Diatrypaceae* based on combined ITS and *tub2* sequence data. The MP and ML bootstrap support values above 70% are shown at the first and second positions, respectively. Thickened branches represent posterior probabilities above 0.95 from the BI. Ex-type strains are in bold, type species are denoted with the superscript “TS” and the disputable type species are denoted with the superscript “TSQ.” Strains from the current study are in blue.

The phylogenetic analysis combined ITS and *tub2* contained 146 ingroup strains with 1,175 characters including gaps (713 for ITS and 462 for *tub2*), of which 471 were constant, 191 variable characters were parsimony-uninformative, and 513 characters were variable and parsimony-informative. The MP analysis resulted 500 parsimonious trees, and the first tree (TL = 3,637, CI = 0.362, RI = 0.771, RC = 0.279) was presented in Figure 1. For BI analyses, the best-fit model of nucleotide evolution was deduced on the AIC (ITS: GTR + I + G; *tub2*: HKY + I + G). Tree topologies of ML and BI analyses did not significantly differ from the MP. Topology of the phylogenetic analyses were similar to the relevant recently published literature (Senwanna et al., 2017; Shang et al., 2017, 2018; Hyde et al., 2019, 2020a; Phookamsak et al., 2019; Dayarathne et al., 2020a; Konta et al., 2020).

Based on phylogenetic analyses, the phylogram included 27 lineages, representing 21 known (*Allocryptovalsa/Eutypella sensu lato*, *Allodiatrype*, *Anthostoma*, *Cryptosphaeria* 1, *Cryptosphaeria* 2, *Cryptovalsa*, *Diatrypasimilis*, *Diatrype sensu stricto*, *Diatrypella* 1, *Diatrypella* 2, *Eutypa sensu lato*, *Eutypella sensu stricto*, *Eutypa sensu stricto*, *Halocryptosphaeria*, *Halocryptovalsa*, *Halodiatrype*, *Monosporascus*, *Neoeutypella*, *Pedumispora*, *Peroneutypa*, *Quaternaria*) and six incertae sedis clades. Eleven lineages are, herein, described as nine new species and two known species belonging to four genera in *Diatrypaceae* (Figure 1).

Some confused taxa were excluded in the current phylogram after the primary analyses. *Diatrype decorticata* (ANM 1498), *Diatrype enteroxantha* (HUEFS 155116), *Diatrype macowaniana* (CBS 214.8), *Diatrype oregonensis* (DCA600), *Diatrype polycocca* (CBS 213.87), *Diatrype prominens* (ATCC MYA-4410), *Diatrype whitmanensis* (CDB011), *Eutypella parasitica* (CBS 210.39), and *Eutypella prunastri* (CBS 277.87) are not the type strains, which have single clade in phylogenetic tree or mixed with in clade of other genera. And the sequence data of *Halocryptovalsa avicenniae* (MAW 2017a) is inconsistent with the position of genus.

Clade 06 (*Diatrypella* 2): This clade comprises nine newly generated strains and other six *Diatrypella* strains with strong statistical supports (MP/ML/BI = 96/99/1) in Figure 1. *Diatrypella hubeiensis* (CFCC 52413) was the basal subclade close to *Da. yunnanensis*. *Diatrypella shennongensis* (CFCC 52414 and CFCC 52415) formed a single clade with high support (MP/ML/BI = 100/100/1). *Diatrypella betulae* (CFCC 52404, CFCC 52405 and CFCC 52406) clustered close to *Da. shennongensis*. *Diatrypella betulicola* (CFCC 52411 and CFCC 52412) also formed a distinct strongly supported clade (MP/ML/BI = 95/96/1). The isolate CFCC 52409 clustered with *Diatrypella favacea* (CBS 198.49, R191, and DL26C), which was recognized as known species.

Clade 09 (*Diatrype sensu stricto*): The type species of *Diatrype*, *D. disciformis* (CBS 205.87 and GB 5815), and other *Diatrype* and *Diatrypella* species grouped with strong statistical supports (MP/ML/BI = 100/97/1) in Figure 1. Our six new strains clustered in this clade as three different subclades *viz. Diatrype quercicola* (CFCC 52418, CFCC 52419, and CFCC 52420), *D. castaneicola* (CFCC 52425 and CFCC 52426) and *D. betulae* (CFCC 52416). *Diatrype betulae* was the basal subclade close to *D. undulata*. *Diatrype castaneicola* was the internal clade with high support (MP/ML/BI = 99/100/1) close to *D. stigma*. *Diatrype quercicola* formed a single clade with high support (MP/ML/BI = 89/98/1) and grouped with *D. virescens* with no support value.

Clade 12 (*Allocryptovalsa*/*Eutypella sensu lato*): This clade comprises *Allocryptovalsa and part Eutypella species* clustered with strong support values (MP/ML/BI = 87/94/1) in Figure 1. Two isolates (CFCC 52433 and CFCC 52434) grouped together with *Eutypella citricola* (HVVIT07 and HVGRF01) with strong support (MP/ML/BI = 100/100/1). The isolates CFCC 52432 formed a separate branch separated from *Eutypella citricola* and *Eutypella vitis* (MP/ML/BI = 95/96/1), which represented a new species *Allocryptovalsa castaneicola*. *Allocryptovalsa castaneae* (CFCC 52427, CFCC 52428, and CFCC 52429) also regarded as a new species with the distinct strongly supported clade (MP/ML/BI = 96/99/1), which was clustered with *Allocryptovalsa cryptovalsoidea*, *A. elaeidis*, *A. polyspora*, and *A. truncata* (MP/ML/BI = 99/100/1).

### Taxonomy

***Allocryptovalsa*** Senwanna, Phookamsak & K.D. Hyde, Mycosphere 8(10): 1839 (2017).

*Type*: *Allocryptovalsa polyspora* Senwanna, Phookamsak & K.D. Hyde, Mycosphere 8(10): 1840 (2017).

*Known distribution*: Australia, China, Germany, India, Thailand, and United States (Saccardo, 1882; Trouillas et al., 2011; Senwanna et al., 2017; Hyde et al., 2020b; Konta et al., 2020; This study).

*Notes*: *Allocryptovalsa* typified with *A. polyspora* was originally introduced to accommodate another two new combination species (*A. cryptovalsoidea* and *A. rabenhorstii*), which was with the character of immersed perithecia, polysporous asci, and allantoid ascospores (Senwanna et al., 2017). Later, Hyde et al. (2020a); Konta et al. (2020) reported *A. elaeidis* and *A. truncata* isolated from *Elaeis guineensis* and decaying twig, respectively. In this study, we introduce two additional species, *Allocryptovalsa castaneae* and *A. castaneicola*, based on morphological coupled with molecular data (Figure 1; clade 12).

**FIGURE 2:**
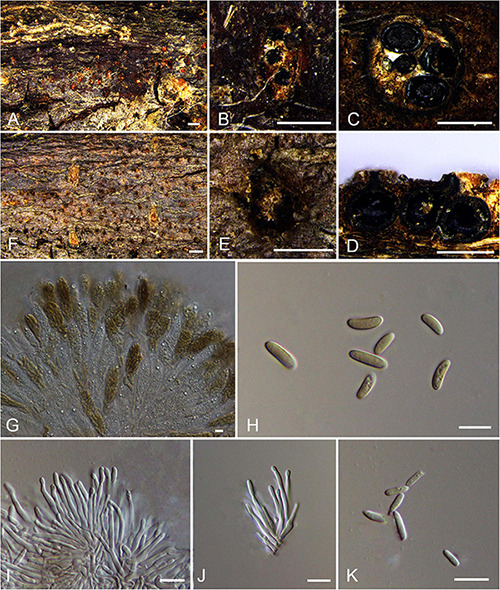
Holomorph of *Allocryptovalsa castaneae* (BJFU CF2020518, holotype). **(A)** Ascomata on the host. **(B)** Ascoma on the host. **(C)** Transverse section of ascoma. **(D)** Longitudinal section through ascoma. **(E)** Conidiomata on the host. **(F)** Conidioma on the host. **(G)** Asci and ascospores. **(H)** Ascospores. **(I,J)** Conidia attatch to conidiogenous cells. **(K)** Conidia. Scale bars: **(A,F)** = 1 mm; **(B−E)** = 500 μm; **(G−K)** = 10 μm.

***Allocryptovalsa castaneae*** N. Jiang & X.L. Fan sp. nov. Figure 2.

MycoBank MB 837777.

*Typification*: CHINA. Hebei Province, Qinhuangdao City, Qinglong County, 119°11′52.25″ E 40°22′52.13″ N, 246 m msl., from branches of *Castanea mollissima*, 16 Oct. 2017, C.M. Tian & N. Jiang, **holotype** BJFU CF2020518, ex-type culture CFCC 52428. Hebei Province, Qinhuangdao City, Qinglong County, 119°11′52.25″ E 40°22′52.13″ N, 246 m msl., from branches of *Castanea mollissima*, 16 Oct. 2017, C.M. Tian & N. Jiang, **isotype** BJM 240506, ex-isotype culture CFCC 52429.

*Etymology*: Named after the host genus from which it was collected, *Castanea*.

*Diagnosis*: Phylogenetically sister to *Allocryptovalsa rabenhorstii*, differs by the smaller size of ascospores (8–11 × 2.5–3.5 vs. 13.5–15 × 4–5 μm).

*Descriptions*: *Necrotrophic* on branches of *Castanea mollissima*. Sexual morph: *Stromata* solitary to gregarious, immersed in the bark, erumpent through the surface of bark, with 3–5 perithecia arranged irregularly (0.3–)0.5–0.8 mm (av. = 0.6 ± 0.2 mm, *n* = 10) in diam. *Ectostromatic disc* orange, unconspicuous, circular to oblong, with 3–5 ostioles arranged irregularly per disc. *Ostioles* numerous, brown to black, at the same level as the disc, scattered (85–)120–130 μm (av. = 122.4 ± 14.0 μm, *n* = 10) in diam. *Perithecia* outer surface lacking powdery entostroma, black, flask-shaped to spherical, with discrete perithecial necks (320–)360–400(−420) μm (av. = 379.7 ± 19.7 μm, *n* = 10) in diam. *Asci* clavate to elongate obovoid, polysporous, thin-walled, short pedicellate, apically rounded (52–)60–83(–92) × (11–)12–17(–25) μm (av. = 71.5 ± 11.4 × 14.4 ± 2 μm, *n* = 30). *Ascospores* elongate-allantoid, thin-walled, pale yellowish to pale brown at maturity, slightly curved, aseptate, 8–11(–13) × 2.5–3.5 (–4) μm (av. = 10.1 ± 0.8 × 3.1 ± 0.4 μm, *n* = 30). Asexual morph: Coelomycetous. *Conidiomata* pycnidial, immersed in the bark, scattered, erumpent through the surface of bark. *Ectostromatic disc* flat or concave, orange, surrounded by bark flaps, circular to ovoid, with 8–10 ostioles arranged circularly on per disc (200–)260–320(–370) μm (av. = 280.8 ± 43.3 μm, *n* = 10) in diam. *Ostioles* black, at the same level as the disc surface (45–)60–70 μm (av. = 64.1 ± 9.8 μm, *n* = 10) in diam. *Conidiogenous cells* holoblastic conidiogenesis, approximately cylindrical, hyaline, integrated, arising from pseudoparenchymatous cells, unicellular, with wide base producing conidia at the apex (15–)19–30(–31) × (1–)1.5–2(–2.5) μm (av. = 24.5 ± 4.9 × 1.7 ± 0.2 μm, *n* = 30). *Conidia* hyaline, elongate-allantoid, not curved, smooth, aseptate (4–)5–7(–8) × (1–) 1.5–2(–2.5) μm (av. = 5.9 ± 0.9 × 1.8 ± 0.2 μm, *n* = 30).

*Culture characteristics*: Cultures are initially white with irregular margin, becoming dark green at the margin and stopping growing with 7 cm in diam. after 2 weeks, comprising dense, irregular, flat mycelium.

*Known host and distribution*: Known on *Castanea mollissima* and *Juglans regia* in China.

*Additional collection examined*: CHINA. Yunnan Province, Chuxiong Yi Autonomous Prefecture, Dayao County, 101°20′15.7″ E 25°44′47.19″ N, 2,002 m msl., from branches of *Juglans regia*, August 07, 2015, N. Zhao, **paratype** BJFU CF2020516, ex-paratype culture CFCC 52427.

*Notes*: Three new strains isolated from branches of *Castanea mollissima* and *Juglans regia*, show high support value (MP/ML/BI = 99/100/1) with the closely clustered isolates in *Allocryptovalsa* (Figure 1; Clade 12: *Allocryptovalsa*/*Eutypella sensu lato*). Moreover, this species has different morphological characters. Morphological comparison of members of *Allocryptovalsa* is provided in Supplementary Table 3. Other species of this genus were not reported with asexual morph. Therefore, *Allocryptovalsa castaneae* showed coelomycetous asexual morph from host in China for the first time. In addition, *Allocryptovalsa castaneae* differs from *A. castaneicola* (from *Castanea mollissima*), *A. cryptovalsoidea* (from *Ficus carica*), *A. elaeidis* (from *Elaeis guineensis*), *A. polyspora* (from *Hevea brasiliensis*), and *A. rabenhorstii* (from *Vitis vinifera* and *Sambuscus nigra*) in host association (Saccardo, 1882; Trouillas et al., 2011; Senwanna et al., 2017; Konta et al., 2020).

**FIGURE 3:**
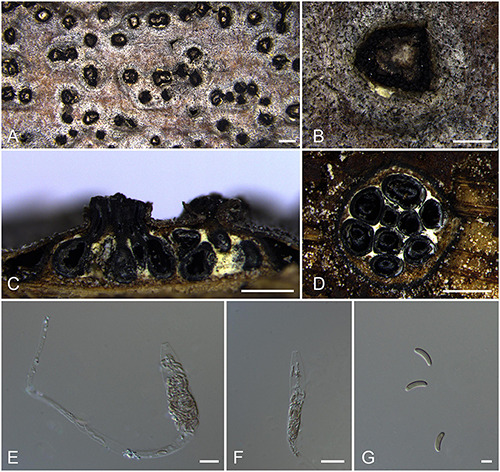
Sexual morph of *Allocryptovalsa castaneicola* (BJFU CF2020519, holotype). **(A)** Ascomata on the host. **(B)** Ascoma on the host. **(C)** Longitudinal section through ascoma. **(D)** Transverse section of ascoma. **(E,F)** Ascus and ascospores. **(G)** Ascospores. Scale bars: **(A)** = 1 mm; **(B–D)** = 500 μm; **(E–G)** = 10 μm.

***Allocryptovalsa castaneicola*** N. Jiang & X.L. Fan sp. nov. Figure 3.

MycoBank MB 837787.

*Typification*: CHINA. Hebei Province, Chengde City, Kuancheng Manchu Nationality Autonomous County, 118°27′54″ E 40°38′37″ N, 450 m msl., from branches of *Castanea mollissima*, 14 Oct. 2017, C.M. Tian & N. Jiang, **holotype** BJFU CF2020519, ex-type culture CFCC 52432. Hebei Province, Chengde City, Kuancheng Manchu Nationality Autonomous County, 118°27′54″ E 40°38′37″ N, 450 m msl., from branches of *Castanea mollissima*, 14 Oct. 2017, C.M. Tian & N. Jiang, **isotype** BJM 240515.

*Etymology*: Named after the host genus from which it was collected, *Castanea*.

*Diagnosis*: *Allocryptovalsa castaneicola* differs from other *Allocryptovalsa* species by its polysporous asci and larger size of ascospores (22–25 × 5–6 μm).

*Descriptions*: *Necrotrophic* on branches of *Castanea mollissima*. Sexual morph: *Stromata* scattered to gregarious, immersed in the bark, erumpent through the surface of bark, with 8–10 perithecia arranged irregularly (1.5–)1.7–2.0 mm (av. = 1.8 ± 0.2 mm, *n* = 10) in diam. *Ectostromatic disc* brown, circular to oblong, with more than 10 ostioles arranged circularly per disc, 0.7–0.9(–1.0) mm (av. = 0.8 ± 0.1 mm, *n* = 10) in diam. *Ostioles* numerous, gregarious, umbilicate, 4-sulcate dark brown to black, at the same level as the disc, 104–120(–140) μm (av. = 114.9 ± 11.3 μm, *n* = 10) in diam. *Perithecia* outer surface coated with yellow, powdery entostromablack, flask-shaped, perithecial necks erumpent in groups (200–)250–320(–380) μm (av. = 282.7 ± 49.8 μm, *n* = 10) in diam. *Asci* clavate to elongate obovoid, polysporous, thin-walled, long pedicellate, apically flat, 194–202 × 15–21 μm (av. = 198.4 ± 3.3 × 18.9 ± 0.5 μm, *n* = 10). *Ascospores* elongate-allantoid, thin-walled, pale yellowish to pale brown at maturity, slightly curved, aseptate, smooth, 22–25 × 5–6 μm (av. = 23.8 ± 1.1 × 5.4 ± 0.3 μm, *n* = 30). Asexual morph: not observed.

*Culture characteristics*: Colonies are initially white, uniform, becoming dark after 2 weeks.

*Known host and distribution*: Known only on *Castanea mollissima* in Hebei Province, China.

*Notes*: The new species displays some features of morphology typical of the recent genus *Allocryptovalsa* (well-developed ascostromata producing polysporous asci and allantoid ascospores) (Senwanna et al., 2017), although it appears closer placed in *Eutypella sensu lato* (Figure 1; Clade 12). Morphologically, *Allocryptovalsa castaneicola* differs from the closest species *Eutypella australiensis* by larger size of asci (194–202 × 15–21 vs. 40–50 × 7–8.5 μm) and ascospores (22–25 × 5–6 vs. 8–10 × 3 μm) (Trouillas et al., 2010a). *Allocryptovalsa castaneicola* was also distinguished from other *Eutypella* species resembles by having polysporous asci rather than the 8-spored asci (Trouillas et al., 2010b). Thus, we introduce it here as a new species in genus *Allocryptovalsa*.

Moreover, *Allocryptovalsa castaneicola* can differ from *Cryptovalsa* species by having a yellow rather than white powdery entostroma appeared on the ascomatal outer surface (Dayarathne et al., 2020b). Also, phylogenetic analyses show affinities of this fungus with strains from *Eutypella* spp. Therefore, the assignment of the strains to the genus *Eutypella sensu lato* (Figure 1; Clade 12) may require future reconsideration.

***Diatrype*** Fr., Summa veg. Scand., Sectio Post. (Stockholm): 384, 1849.

*Type*: *Diatrype disciformis* (Hoffm.) Fr., Summa veg. Scand., Sectio Post. (Stockholm): 385 (1849).

*Known distribution*: Asia, Europe, North America, Oceania, and South Africa (Doidge, 1950; Munk, 1957; Conners, 1967; Rappaz, 1987; Mulenko et al., 2008; Trouillas et al., 2010a, b).

*Notes*: The genus *Diatrype* was established by Fries (1849) with *Diatrype disciformis* as the generic type, which have often been regarded as saprobes on decaying wood and have a strong ability to resist harsh conditions (Senanayake et al., 2015). Due to the taxonomic confusion, *Diatrype* may require a thorough revision together with the entire family in the future.

**FIGURE 4:**
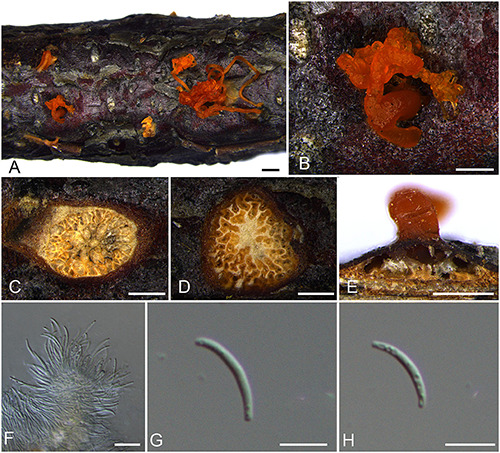
Asexual morph of *Diatrype betulae* (BJFU CF2020510, holotype). **(A)** Conidiomata on the host. **(B)** Conidioma on the host. **(C,D)** Transverse section of conidioma. **(E)** Longitudinal section through conidioma. **(F)** Conidiogenous cells. **(G,H)** Conidia. Scale bars: **(A)** = 1 mm; **(B−E)** = 500 μm; **(F−H)** = 10 μm.

***Diatrype betulae*** H.Y. Zhu & X.L. Fan sp. nov. Figure 4.

MycoBank MB 837784.

*Typification*: CHINA. Beijing City, Mentougou District, Mount Dongling, Xiaolongmen Forestry Centre, 115°26′51.27″ E 39°58′19.62″ N, 1,302 m msl., from branches of *Betula davurica*, 21 Aug. 2017, H.Y. Zhu & X.L. Fan, **holotype** BJFU CF2020510, ex-type culture CFCC 52416. Beijing City, Mentougou District, Mount Dongling, Xiaolongmen Forestry Centre, 115°26′51.27″ E 39°58′19.62″ N, 1,302 m msl., from branches of *Betula davurica*, 21 Aug. 2017, H.Y. Zhu & X.L. Fan, **isotype** BJM 240512.

*Etymology*: Named after the host genus from which it was collected, *Betula*.

*Diagnosis*: Phylogenetically, *Diatrype betulae* formed a separate clade.

*Descriptions*: *Necrotrophic* on branches of *Betula davurica*. Sexual morph: not observed. Asexual morph: Coelomycetous. *Conidiomata* pycnidial, immersed in the bark, scattered, erumpent slightly through the surface of bark, with multiple locules and orange colloid conidial drops exuding from the ostioles. Locules numerous, buff, circular to ovoid, 1.0–1.4 mm (av. = 1.2 ± 0.2 mm, *n* = 10) in diam. *Conidiogenous cells* approximately cylindrical, mostly straight, discrete or integrated, arising from pseudoparenchymatous cells, hyaline, unicellular, with wide base producing conidia at the apex, holoblastic conidiogenesis (12–)14–20 × 1–2 μm (av. = 16.6 ± 3.5 × 1.2 ± 0.2 μm, *n* = 30). *Conidia* hyaline, filiform, smooth or rough, aseptate, 10–13 × 1–2 μm (av. = 11.7 ± 1.2 × 1.5 ± 0.1 μm, *n* = 30).

*Culture characteristics*: Cultures are white, uniform, dense, slow growing, reaching 4 cm after 2 weeks, not produced pigmentation on PDA media.

*Known host and distribution*: Known only on *Betula davurica* in Beijing City, China.

*Notes*: *Diatrype betulae* was isolated from branches of *Betula davurica* in Beijing, China. One strain of *Diatrype betulae* (CFCC 52416) clusters as a single lineage (Figure 1). *Diatrype albopruinosa*, *D. undulata*, and *D. stigma* were also reported from *Betula* sp. (Tiffany and Gilman, 1965; Chlebicki, 2005; Mulenko et al., 2008; Goos, 2010). However, *Diatrype betulae* can be easily distinguished from the other three. *Diatrype albopruinosa* and *D. undulata* lack the asexual morph and sequences of *D. albopruinosa* are unavailable. *Diatrype stigma* was known on various hosts with worldwide distribution, but it can differ from *D. betulae* by smaller conidia (4.5–7.5 × 1–2 vs. 10–13 × 1–2 μm) (Rappaz, 1987). *Diatrype betulae* is also phylogenetically closely related to *D. bullata*, *D. castaneicola*, *D. disciformis*, *D. iranensis*, *D. macrospora*, *D. quercicola*, *D. quercina*, *D. spilomea*, and *D. virescens*. *Diatrype betulae* can differ from *D. bullata* that is a common species isolated from willows in the northern hemisphere in host association (Vasilyeva and Ma, 2014). *Diatrype iranensis*, *D. macrospora*, *D. quercicola*, and *D. quercina* were only reported from *Quercus* sp. (Croxall, 1950; Mehrabi et al., 2015, 2016). *Diatrype betulae* can easily be distinguished from them by its plant host (*Betula* sp.) and smaller conidia (18–38 × 0.6–0.8 μm in *D. iranensis* and 20–40 × 0.6–0.8 μm in *D. macrospora*) (Mehrabi et al., 2015, 2016). In morphology, *D. betulae* can be differentiated from *D. disciformis* and *D. virescens* by having asexual morph. Moreover, *D. betulae* differs from other closest species *D. castaneicola* and *D. spilomea* by the size of conidia (10–13 × 1–2 vs. 4–6 × 1–1.5, 13–18.5 × 1–1.2 μm) (Rappaz, 1987).

**FIGURE 5:**
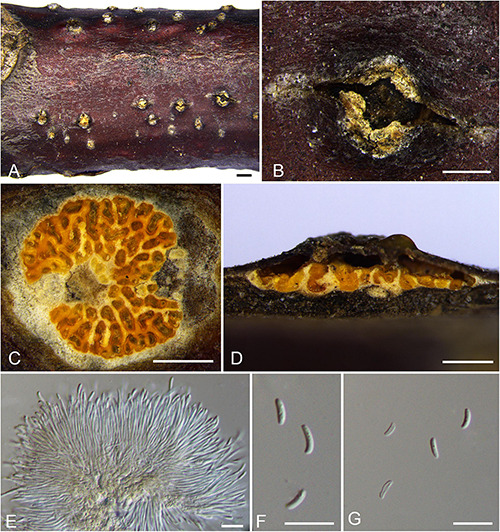
Asexual morph of *Diatrype castaneicola* (BJFU CF2020515, holotype). **(A)** Conidiomata on the host. **(B)** Conidioma on the host. **(C)** Transverse section of conidioma. **(D)** Longitudinal section through conidioma. **(E)** Conidiogenous cells. **(F,G)** Conidia. Scale bars: **(A)** = 1 mm; **(B–D)** = 500 μm; **(E–G)** = 10 μm.

***Diatrype castaneicola*** N. Jiang & X.L. Fan sp. nov. Figure 5.

MycoBank MB 837785.

*Typification*: CHINA. Hebei Province, Qinhuangdao City, Qinglong County, 119°11′52.25″ E 40°22′52.13″ N, 246 m msl., from branches of *Castanea mollissima*, 16 Oct. 2017, C.M. Tian & N. Jiang, **holotype** BJFU CF2020515, ex-type culture CFCC 52425. Hebei Province, Qinhuangdao City, Qinglong County, 119°11′52.25″ E 40°22′52.13″ N, 246 m msl., from branches of *Castanea mollissima*, 16 Oct. 2017, C.M. Tian & N. Jiang, **isotype** BJM 240513, ex-isotype culture CFCC 52426.

*Etymology*: Named after the host genus from which it was collected, *Castanea*.

*Diagnosis*: Phylogenetically, *Diatrype castaneicola* formed a separate clade.

*Descriptions*: *Necrotrophic* on branches of *Castanea mollissima*. Sexual morph: not observed. Asexual morph: Coelomycetous. *Conidiomata* pycnidial, immersed in the bark, scattered, erumpent slightly through the surface of bark, with multiple locules (0.7–)0.8–1.2(–1.6) mm (av. = 1.0 ± 0.2 mm, *n* = 10). *Ectostromatic disc* brown, unconspicuous, circular to ovoid. Ostiole unconspicuous, gray to black, at the same the level as the disc surface, covered by ectostroma tissue. Locules numerous, circular to ovoid, 1.0–1.4 mm (av. = 1.2 ± 0.2 mm, *n* = 10) in diam. *Conidiogenous cells* approximately cylindrical, mostly straight, discrete or integrated, arising from pseudoparenchymatous cells, hyaline, unicellular, with wide base producing conidia at the apex, holoblastic conidiogenesis (15–)18–26(–33) × 1–1.5 μm (av. = 22.5 ± 3.5 × 1.2 ± 0.2 μm, *n* = 30). *Conidia* hyaline, elongate-allantoid, slightly curved, smooth, aseptate, multiguttulate, often containing guttules per cell, 4–6 × 1–1.5 μm (av. = 5.3 ± 0.6 × 1.3 ± 0.2 μm, *n* = 30).

*Culture characteristics*: Colonies are white, dense, not produced pigmentation on PDA media. Pycnidia distributed irregularly on colony surface with yellow cream conidial drops exuding from the ostioles.

*Known host and distribution*: Known only on *Castanea mollissima* in Hebei Province, China.

*Notes*: *Diatrype castaneicola* was isolated from branches of *Castanea mollissima* in Hebei Province, China. Our new isolates (CFCC 52425 and CFCC 52426) grouped in *Diatrype sensu stricto* as a separate clade with high statistical support (MP/ML/BI = 99/100/1) (Figure 1). *Diatrype castaneicola* differs from the closely related one, *D. stigma*, by its smaller conidia (4–6 × 1–1.5 vs. 4.5–7.5 × 1–2 mm) (Rappaz, 1987). Currently, *Diatrype castaneicola* is reported with only the asexual morph, thus more studies are essential to report the sexual morph.

**FIGURE 6:**
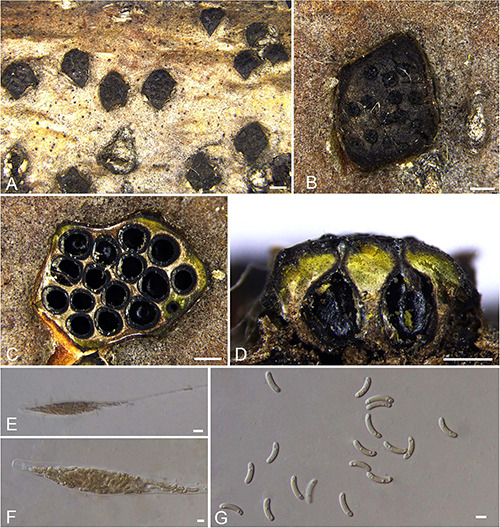
Sexual morph of *Diatrype quercicola* (BJFU CF2020512, holotype). **(A)** Ascomata on the host. **(B)** Ascoma on the host. **(C)** Transverse section of ascoma. **(D)** Longitudinal section through ascoma. **(E,F)** Ascus and ascospores. **(G)** Ascospores. Scale bars: **(A)** = 1 mm; **(B–D)** = 500 μm; **(E–G)** = 10 μm.

***Diatrype quercicola*** H.Y. Zhu & X.L. Fan sp. nov. Figure 6.

MycoBank MB 837786.

*Typification*: CHINA. Beijing City, Mentougou District, Mount Dongling, Xiaolongmen Forestry, 115°26′51.27″ E 39°58′19.62″ N, 1,267 m msl., from branches of *Quercus mongolica*, 21 Aug. 2017, H.Y. Zhu & X.L. Fan, **holotype** BJFU CF2020512, ex-type culture CFCC 52418. Beijing City, Mentougou District, Mount Dongling, Xiaolongmen Forestry, 115°26′51.27″ E 39°58′19.62″N, 1,267 m msl., from branches of *Quercus mongolica*, 21 Aug. 2017, H.Y. Zhu & X.L. Fan, **isotype** BJM 240514, ex-isotype culture CFCC 52419.

*Etymology*: Named after the host genus from which it was collected, *Quercus*.

*Diagnosis*: Phylogenetically, *Diatrype quercicola* formed a separate clade. Howeverits asci are polysporous and differ from the common 8-ascospores asci in *Diatrype*.

*Descriptions*: *Necrotrophic* on branches of *Quercus mongolica*. Sexual morph: *Stromata* solitary, immersed in the bark, erumpent through the surface of bark, with more than 10 perithecia arranged irregularly (2.3–)2.5–2.9 mm (av. = 2.7 ± 0.2 mm, *n* = 10) in diam. *Ectostromatic disc* brown, circular to oblong, with more than 10 ostioles arranged regularly per disc (1.5–)1.7–2.4 mm (av. = 2.0 ± 0.3 mm, *n* = 10) in diam. *Ostioles* dark brown to black, at the same level as the disc, scattered, 210–275(–335) μm (av. = 253.3 ± 22.1 μm, *n* = 10) in diam. *Perithecia* outer surface coated with yellow, powdery entostromablack, flask-shaped, with discrete perithecial necks (475–)515–620(–665) μm (av. = 566.6 ± 53.2 μm, *n* = 10) in diam. *Asci* clavate to elongate obovoid, polysporous, thin-walled, long pedicellate, apically rounded, 172–183 × (16–)20–43 μm (av. = 178 ± 3.3 × 31.7 ± 10.9 μm, *n* = 10). *Ascospores* elongate-allantoid, thin-walled, pale yellowish to pale brown at maturity, slightly curved, aseptate, multiguttulate, often containing 1–3 symmetrical guttules per cell, 17–27 × 4–6 μm (av. = 22.6 ± 2.6 × 5.4 ± 0.6 μm, *n* = 30). Asexual morph: not observed.

*Culture characteristics*: Colonies are white, irregular, reaching 9 cm after 7 days, not produced pigmentation on PDA media.

*Known host and distribution*: Known only on *Quercus mongolica* in Beijing City, China.

*Additional collection examined*: CHINA. Beijing City, Mentougou District, Mount Dongling, Xiaolongmen Forestry, 115°26′51.27″ E 39°58′19.62″N, 1,267 m msl., from branches of *Quercus mongolica*, 21 Aug. 2017, H.Y. Zhu & X.L. Fan, BJFU CF2020513, living culture CFCC 52420.

*Notes*: *Diatrype quercicola* is a unique *Diatrype* species isolated from *Quercus mongolica* in China. Asci of this species are polysporous and differ from the common 8-ascospores asci of *Diatrype*, including the closely related taxa, *D. virescens*. Moreover, *D. quercicola* can differ from *D. virescens* by larger asci and ascospres (172–183 × 20–43 vs. 35–40 × 4–6 μm; 17–27 × 4–6 vs. 12–14 × 2.5–3 μm) (Vasilyeva and Stephenson, 2005). Nevertheless, based on phylogeny analyses, this taxon appears best placed in *Diatrype*. Therefore, the assignment of this species to *Diatrype* may require reconsideration due to the taxonomic confusion around *Diatrypaceae*.

*Diatrype albopruinosa*, *D. standleyi*, and *D. stigmaoides* were also isolated from *Quercus* sp. (Tiffany and Gilman, 1965; Rappaz, 1987; Mendez-Mayboca et al., 2008; Vasilyeva and Stephenson, 2009). *Diatrype albopruinosa* and *D. standleyi* differ from *D. quercicola* by its 8-ascospores asci (Vasilyeva and Ma, 2014). *Diatrype stigmaoides* differs from *D. quercicola* by its hyaline ascospores (Vasilyeva and Stephenson, 2009).

***Diatrypella*** (Ces. & De Not.) Nitschke, Pyr. Germ: 69, 1867.

*Type*: *Diatrypella verruciformis* (Ehrh.) Nitschke, Pyrenomyc. Germ. 1: 76, 1867 [Current name: *Diatrypella favacea* (Fr.)].

*Known distribution*: Asia, Europe, North America, Oceania, South Africa, and South America (Unamuno, 1941; Doidge, 1950; Rao, 1966; Conners, 1967; Hanlin, 1992; Crous et al., 2016).

*Notes*: *Diatrypella* was introduced by Nitschke (1867) to accommodate *Diatrype* sect. *Diatrypella* Ces. & De Not. (1863). The type species is disputable that Croxall (1950) relegated *Da. verruciformis* (as *D. verrucaeformis*) to synonymy with *Da. favacea*, whereas Munk (1957) recognized both species as different fungi appear to have been frequently included under *Da. verruciformis*. Glawe and Rogers (1984) believed *Diatrypella* was well distinguished genus as its well-delveloped stromata and the single host affiliation (*Da. verruciformis* on *Alnus* and *Da. favacea* on *Betula*). Further studies are needed to clarify them. Vasilyeva and Stephenson (2005) pointed out that *Diatrypella* morphologically resembled *Cryptovalsa. Diatrypella* and *Cryptovalsa* were mentioned as the polysporous complement of *Diatrype* and *Eutypa* (Vasilyeva and Stephenson, 2005). Nevertheless, it is still difficult to determine the differences between *Diatrypella* and *Cryptovalsa* based on morphological characters (Acero et al., 2004; Vasilyeva and Stephenson, 2005). Therefore, multilocus phylogeny including more representative taxa are needed to clarify the relationship among species in *Diatrypella* (Mehrabi et al., 2015).

Newly generated nine isolates show affinities to *Diatrypella favacea* clade based on phylogenetic analyses. Therefore, we prefer the assignment of the strains to the genus *Diatrypella* (Figure 1; Clade 06) preliminarily and tentatively, as it may require future reconsideration after the typification work on type species of *Diatrypella*.

**FIGURE 7:**
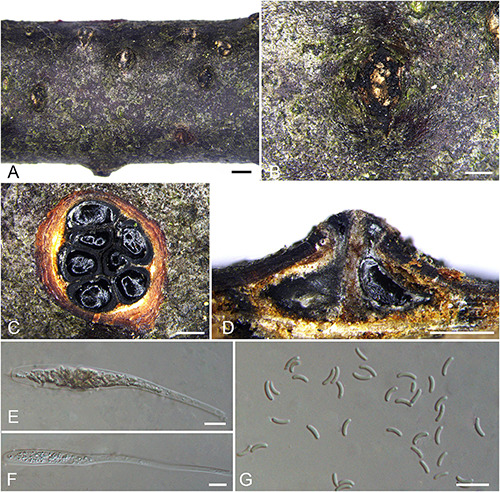
Sexual morph of *Diatrypella betulae* (BJFU CF2020501, holotype). **(A)** Ascomata on the host. **(B)** Ascoma on the host. **(C)** Transverse section of ascoma. **(D)** Longitudinal section through ascoma. **(E)** Ascus and ascospores. **(F)** ascus. **(G)** Ascospores. Scale bars: **(A)** = 1 mm; **(B–D)** = 500 μm; **(E–G)** = 10 μm.

***Diatrypella betulae*** H.Y. Zhu & X.L. Fan sp. nov. Figure 7.

MycoBank MB 837778.

*Typification*: CHINA. Hubei Province, Shennongjia Forest District, Shennong Stream, 110°17′51.54″ E 31°28′15.79″ N, 2273 m msl., from branches of *Betula albosinensis*, 17 Aug. 2017, Z. Du & Q. Yang, **holotype** BJFU CF2020501, ex-type culture CFCC 52406. Hubei Province, Shennongjia Forest District, Shennong Stream, 110°17′51.54″ E 31°28′15.79″ N, 2273 m msl., from branches of *Betula albosinensis*, 17 Aug. 2017, Z. Du & Q. Yang, **isotype** BJM 240507, ex-isotype culture CFCC 52404.

*Etymology*: Named after the host genus from which it was collected, *Betula*.

*Diagnosis*: Phylogenetically sister to *Diatrypella shennongensis*, differ by the number of perithecia.

*Descriptions*: *Necrotrophic* on branches of *Betula albosinensis*. Sexual morph: *Stromata* solitary, immersed in the bark, erumpent through the surface of bark, with 7–10 perithecia arranged irregularly (1.0–)1.6–2.1 mm (av. = 1.8 ± 0.2 mm, *n* = 10) in diam. *Ectostromatic disc* orange, circular to oblong, with 7–10 ostioles arranged regularly per disc, 0.8–1.3(–1.5) mm (av. = 1.0 ± 0.3 mm, *n* = 10) in diam. *Ostioles* numerous, scattered, umbilicate, sulcate, dark brown to black, at the same level as the disc (125–)175–240 μm (av. = 190.4 ± 38.0 μm, *n* = 10) in diam. *Perithecia* outer surface lacking powdery entostroma, black, flask-shaped, with long discrete perithecial necks (430–)530–830(–870) μm (av. = 680.2 ± 152.7 μm, *n* = 10) in diam. *Asci* clavate to elongate obovoid, polysporous, thin-walled, long pedicellate, apically rounded to flat, 132–140 × 7.5–10.5(–11.5) μm (av. = 136.2 ± 3 × 9.5 ± 1.4 μm, *n* = 10). *Ascospores* elongate-allantoid, thin-walled, pale yellowish to pale brown at maturity, slightly curved, smooth, aseptate (4.5–)5–7 × 1–2 μm (av. = 5.7 ± 0.5 × 1.5 ± 0.2 μm, *n* = 30). Asexual morph: not observed.

*Culture characteristics*: Cultures are flat, reaching 9 cm diam. after 7–10 days. Colonies white, rough on surface, not produced pigmentation on PDA media.

*Known host and distribution*: Known only on *Betula albosinensis* in Hubei Province, China.

*Additional collection examined*: CHINA. Hubei Province, Shennongjia Forest District, Shennong Stream, 110°17′51.54″ E 31°28′15.79″ N, 2,273 m msl., from branches of *Betula albosinensis*, 17 Aug. 2017, Z. Du & Q. Yang, BJFU CF2020502, living culture CFCC 52405.

*Notes*: Three strains representing *Diatrypella betulae* appear most closely related to *Da. shennongensis* reported from the same host plant *Betula* sp., which also clustered in a well-supported clade (MP/ML/BI = 92/100/1). In morphology of asci and ascospores, *Diatrypella betulae* resembles *Da. shennongensis* by the size of asci and ascospores (132–140 × 7.5–10.5 vs. 129–140 × 8–12; 5–7 × 1–2 vs. 5–6.5 × 1–1.5 μm), but they can be distinguished by the number of perithecia (less than 10 vs. more than 10). Moreover, *Diatrypella betulae* differs from *Da. shennongensis* based on ITS and *tub2* loci (67/665 in ITS and 13/416 in *tub2*).

**FIGURE 8:**
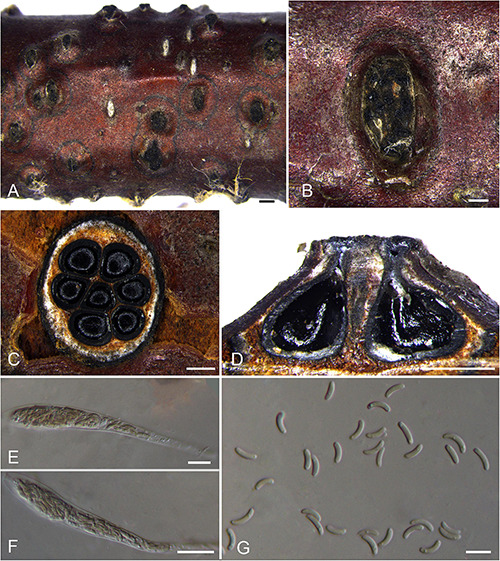
Sexual morph of *Diatrypella betulicola* (BJFU CF2020505, holotype). **(A)** Ascomata on the host. **(B)** Ascoma on the host. **(C)** Transverse section of ascoma. **(D)** Longitudinal section through ascoma. **(E,F)** Ascus and ascospores. **(G)** Ascospores. Scale bars: **(A)** = 1 mm; **(B–D)** = 500 μm; **(E–G)** = 10 μm.

***Diatrypella betulicola*** H.Y. Zhu & X.L. Fan sp. nov. Figure 8.

MycoBank MB 837779.

*Typification*: CHINA. Beijing City, Mentougou District, Mount Dongling, Xiaolongmen Forestry Centre, 115°26′51.27″ E 39°58′19.62″ N, 1,209 m msl., from branches of *Betula davurica*, 21 Aug. 2017, H.Y. Zhu & X.L. Fan, **holotype** BJFU CF2020505, ex-type culture CFCC 52411. Beijing City, Mentougou District, Mount Dongling, Xiaolongmen Forestry Centre, 115°26′51.27″ E 39°58′19.62″ N, 1,209 m msl., from branches of *Betula davurica*, 21 Aug. 2017, H.Y. Zhu & X.L. Fan, **isotype** BJM 240508.

*Etymology*: Named after the host genus from which it was collected, *Betula*.

*Diagnosis*: *Diatrypella betulicola* is different from other species of *Diatrypella* on host association and the size of asci and ascospores.

*Descriptions*: *Necrotrophic* on branches of *Betula davurica* and *B. platyphylla*. Sexual morph: *Stromata* solitary, immersed in the bark, erumpent through the surface of bark, with 7–10 perithecia arranged regularly (1.3–)1.5–1.9(–2.1) mm (av. = 1.7 ± 0.2 mm, *n* = 10) in diam. *Ectostromatic disc* brown to black, circular to oblong, with more than 10 ostioles arranged regularly per disc (0.5–)0.6–1.2(–1.4) mm (av. = 0.9 ± 0.3 mm, *n* = 10) in diam. *Ostioles* numerous, scattered, umbilicate, sulcate, dark brown to black, at the same level as the disc (145–)185–240(–270) μm (av. = 211.0 ± 31.1 μm, *n* = 10) in diam. *Perithecia* outer surface lacking powdery entostroma, black, flask-shaped, with short discrete perithecial necks (520–)600–730(–790) μm (av. = 667.8 ± 61.1 μm, *n* = 10) in diam. *Asci* clavate to elongate obovoid, polysporous, thin-walled, long pedicellate, apically flat,117–133 × 10–12 μm (av. = 124.9 ± 7.7 × 10.6 ± 0.5 μm, *n* = 10). *Ascospores* elongate-allantoid, thin-walled, pale yellowish to pale brown at maturity, slightly curved, aseptate, smooth or multiguttulate, occasionally containing one guttule per cell, 5–8 × 1–2 μm (av. = 6.6 ± 0.6 × 1.6 ± 0.2 μm, *n* = 30). Asexual morph: not observed.

*Culture characteristics*: Cultures are fluffy, reaching 9 cm after 7 days, becoming pale yellow at the margin after 2 weeks. Colonies dense with aerial mycelium at the center, sparse at the margin.

*Known host and distribution*: Known on *Betula davurica* and *B. platyphylla* in Beijing City, China.

*Additional collection examined*: CHINA. Beijing City, Mentougou District, Mount Dongling, Xiaolongmen Forestry, 115°26′51.27″ E 39°58′19.62″ N, 1,209 m msl., from branches of *Betula platyphylla*, 21 Aug. 2017, H.Y. Zhu & X.L. Fan, **paratype** BJFU CF2020506, ex-paratype culture CFCC 52412.

*Notes*: *Diatrypella betulicola* was isolated from branches of *Betula davurica* and *Betula platyphylla* in China. Phylogenetically, two strains representing *Diatrypella betulicola* cluster in a well-supported clade (MP/ML/BI = 95/96/1) in *Diatrypella* 2 clade (Figure 1). *Diatrypella betulae*, *Da. favacea*, and *Da. shennongensis* are the most closely related species. *Diatrypella betulicola* can be differentiated from *Da. betulicola* and *Da. favacea* by the number of ostioles (more than 10 ostioles in *Diatrypella betulae* and less than 10 ostioles in *Da. betulicola* and *Da. favacea*). Moreover, *Da. betulicola* can be easily distinguished from *Da. shennongensis* by unique size of asci (117–133 × 10–12 vs. 129–140 × 8–12 μm) and ascospore (5–8 × 1–2 vs. 5–6.5 × 1–1.5 μm). Moreover, it is different from other species of *Diatrypella* on host association and the size of asci and ascospores (Supplementary Table 4).

**FIGURE 9:**
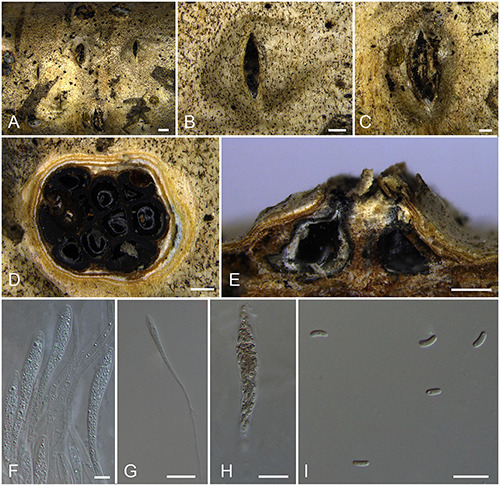
Sexual morph of *Diatrypella favacea* (BJFU CF2020504). **(A)** Ascomata on the host. **(B,C)** Ascoma on the host. **(D)** Transverse section of ascoma. **(E)** Longitudinal section through ascoma. **(F)** Asci. **(G)** Ascus. **(H)** Ascus and ascospores. **(I)** Ascospores. Scale bars: **(A)** = 1 mm; **(B–E)** = 500 μm; **(F–I)** = 10 μm.

***Diatrypella favacea*** (Fr.) Ces. & De Not., Sfer. Ital.: 29 (1863). Figure 9.

*Descriptions*: *Necrotrophic* on branches of *Betula platyphylla*. Sexual morph: *Stromata* solitary, consisting of an inconspicuous pale yellow ectostromatic disc, immersed in the bark, erumpent through the surface of bark, with 7–10 perithecia arranged irregularly (1.9–)2.9–3.3 mm (av. = 3.1 ± 0.2 mm, *n* = 10) in diam. *Ectostromatic disc* brown to black, circular to oblong, with 5–7 ostioles arranged irregularly per disc (0.5–)1.8–2.3(–2.4) mm (av. = 2.1 ± 0.2 mm, *n* = 10) in diam. *Ostioles* numerous, gregarious, umbilicate, sulcate, dark brown to black, at the same level as the disc (230–)240–260(–280) μm (av. = 252.4 ± 16.6 μm, *n* = 10) in diam. Paraphyses elongate cylindrical, 118–125 × 2–3.5 μm (av. = 121.6 ± 2.7 × 2.7 ± 0.7 μm, *n* = 10). *Perithecia* outer surface lacking powdery entostroma, black, flask-shaped to spherical, with long discrete perithecial necks (690–)780–850(940) μm (av. = 821.9 ± 34.1 μm, *n* = 10) in diam. *Asci* clavate to elongate obovoid, polysporous, thin-walled, long pedicellate, apically rounded, 64–124 × (9–)9.5–12(–12.5) μm (av. = 92.7 ± 14.5 × 10.3 ± 1.7 μm, *n* = 10). *Ascospores* short-allantoid, thin-walled, pale yellowish to pale brown at maturity, slightly curved, aseptate, multiguttulate, often containing one guttulae per cell (3.5–)4–5.5(–6) × 1.5–2 μm (av. = 4.8 ± 0.5 × 1.8 ± 0.2 μm, *n* = 30). Asexual morph: not observed.

*Culture characteristics*: Cultures are white, uniform, attaining 9 cm in 7 days. Colonies sparse at the center, medium dense at the margin, rough on the surface, not produced pigmentation on PDA media.

*Known host and distribution*: Known on various hosts with worldwide distribution^3^.

*Collection examined*: CHINA. Xinjiang Uygur Autonomous Region, Bortala Mongol Autonomous Prefecture, Wenquan County, 81°46′22.96″ E 45°13′08.47″ N, 1,439 m msl., from branches of *Betula platyphylla*, July 15, 2017, C.M. Tian & R. Ma, BJFU CF2020504, living culture CFCC 52409.

*Notes*: *Diatrypella favacea* was reported to be restricted to *Betula* spp. in previous study (Saccardo, 1882; Glawe and Rogers, 1984), but then it got involved in the problematic species concept and delimitation with *Da. verruciformis* (Farr et al., 1989). *Diatrypella favacea* and *Da. pulvinata* clustered in a single clade until Hyde et al. (2020b) reported *Da. yunnanensis*. The strain CFCC 52409 clusters with *Diatrypella favacea* (CBS 198.49, DL26C, and R191) in a separate lineage. Morphologically, our strain is similar to those previously reported in terms of the size of asci (64–124 × 9.5–12 vs. 70–90 × 8–12 μm) and ascospores (4–5.5 vs. 6–8 μm) (Vasilyeva and Stephenson, 2005). The current definition of *Diatrypella favacea* seems to be difficult due to the lack of type material with available living culture or DNA sequence data. Thus, the current identification is preliminary and awaits further studies of typification.

**FIGURE 10:**
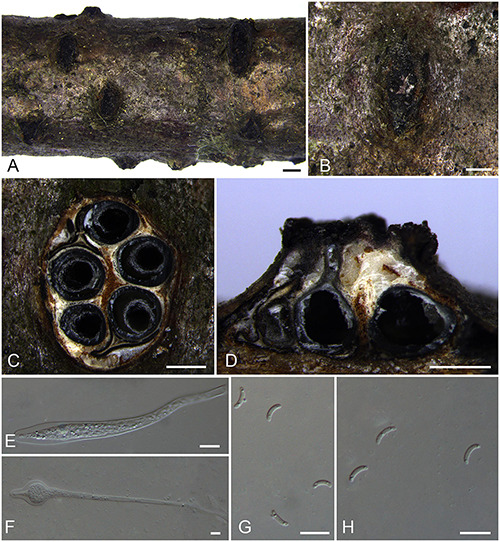
Sexual morph of *Diatrypella hubeiensis* (BJFU CF2020507, holotype). **(A)** Ascomata on the host. **(B)** Ascoma on the host. **(C)** Transverse section of ascoma. **(D)** Longitudinal section through ascoma. **(E,F)** ascus. **(G, H)** Ascospores. Scale bars: **(A)** = 1 mm; **(B–D)** = 500 μm; **(E–H)** = 10 μm.

***Diatrypella hubeiensis*** H.Y. Zhu & X.L. Fan sp. nov. Figure 10.

MycoBank MB 837781.

*Typification*: CHINA. Hubei Province, Shennongjia Forest District, Tianyan Scenic Area, 110°27′36.71″ E 31°42′59.10″ N, 2,140 m msl., from branches of *Betula davurica*, 16 Aug. 2017, Z. Du & Q. Yang, **holotype** BJFU CF2020507, ex-type culture CFCC 52413. Hubei Province, Shennongjia Forest District, Tianyan Scenic Area, 110°27′36.71″ E 31°42′59.10″ N, 2,140 m msl., from branches of *Betula davurica*, 16 Aug. 2017, Z. Du & Q. Yang, **isotype** BJM 240510.

*Etymology*: Named after the location where it was collected, Hubei Province.

*Diagnosis*: Phylogenetically sister to *Diatrypella yunnanensis*, differ by smaller size of ascospores (6–8.5 × 1–2 vs. 18–22 × 3–4 μm).

*Descriptions*: *Necrotrophic* on branches of *Betula davurica*. Sexual morph: *Stromata* solitary, immersed in the bark, erumpent through the surface of bark, with 5–7 perithecia arranged regularly (1.4–)1.7–2.5(–3.0) mm (av. = 2.1 ± 0.4 mm, *n* = 10) in diam. *Ectostromatic disc* brown to black, circular to oblong, with 5–7 ostioles arranged irregularly per disc (0.9–)1.0–2.0(–2.6) (av. = 1.5 ± 0.4 mm, *n* = 10) in diam. *Ostioles* numerous, gregarious, umbilicate, sulcate, dark brown to black, at the same level as the disc (120–)130–170(–200) μm (av. = 143.1 ± 30.4 μm, *n* = 10) in diam. *Perithecia* outer surface lacking powdery entostroma, black, flask-shaped to spherical, with long discrete perithecial necks, 500–680(–720) μm (av. = 619.3 ± 71.3 μm, *n* = 10) in diam. *Asci* clavate to elongate obovoid, occasionally similar to an inverted volumetric flask, polysporous, thin-walled, long pedicellate, apically rounded to flat, 189–240 × 18–21 μm (av. = 213.4 ± 13.3 × 19.8 ± 0.5 μm, *n* = 10). *Ascospores* elongate-allantoid, thin-walled, slightly curved, aseptate, multiguttulate, often containing two symmetrical guttules per cell, 6–8.5(–9) × 1–2 μm (av. = 7.4 ± 0.7 × 1.6 ± 0.2 μm, *n* = 30). Asexual morph: not observed.

*Culture characteristics*: Cultures are white, fluffy, fast growing, attaining 9 cm in 7 days. Colonies dense, slightly raised with aerial mycelium, not produced pigmentation on PDA media.

*Known host and distribution*: Known only on *Betula davurica* in Hubei Province, China.

*Notes*: The only strain CFCC 52413 representing *Diatrypella hubeiensis* clusters with *Da. pulvinata* and *Da. yunnanensis*. However, *Diatrypella pulvinata* was introduced as an asexual fungus in *Quercus garryana. Diatrypella hubeiensis* differs from its closest relative *Da. yunnanensis* by larger asci (189–240 × 18–21 vs. 105–210 × 15–30 μm) and smaller size of ascospores (6–8.5 × 1–2 vs. 18–22 × 3–4 μm) (Hyde et al., 2020b).

**FIGURE 11:**
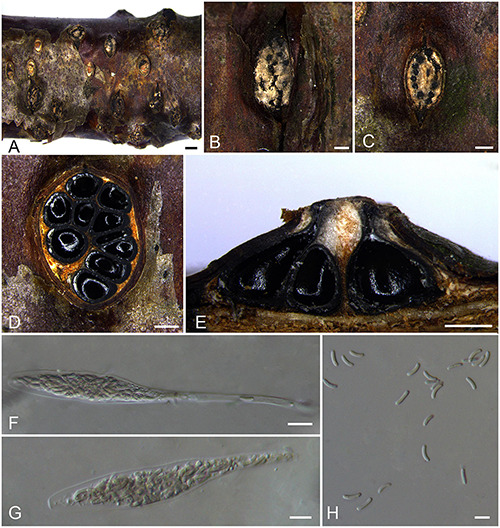
Sexual morph of *Diatrypella shennongensis* (BJFU CF2020508, holotype). **(A)** Ascomata on the host. **(B,C)** Ascoma on the host. **(D)** Transverse section of ascoma. **(E)** Longitudinal section through ascoma. **(F,G)** Ascus and ascospores. **(H)** Ascospores. Scale bars: **(A)** = 1 mm; **(B–E)** = 500 μm; **(F–H)** = 10 μm.

***Diatrypella shennongensis*** H.Y. Zhu & X.L. Fan sp. nov. Figure 11.

MycoBank MB 837780.

*Typification*: CHINA. Hubei Province, Shennongjia Forest District, Shennong Ding, Shennong Camp, 110°17′28.39″ E 31°26′34.59″ N, 2,647 m msl., from branches of *Betula albosinensis*, 17 Aug. 2017, Z. Du & Q. Yang, **holotype** BJFU CF2020508, ex-type culture CFCC 52415. Hubei Province, Shennongjia Forest District, Shennong Ding, Shennong Camp, 110°17′28.39″ E 31°26′34.59″ N, 2,647 m msl., from branches of *Betula albosinensis*, 17 Aug. 2017, Z. Du & Q. Yang, **isotype** BJM 240509, ex-isotype culture CFCC 52414.

*Etymology*: Named after the location where it was collected, Shennong Ding.

*Diagnosis*: Phylogenetically, sister to *Diatrypella betulae*, differ by ITS and *tub2* loci (67/665 in ITS and 13/416 in *tub2*).

*Descriptions*: *Necrotrophic* on branches of *Betula albosinensis*. Sexual morph: *Stromata* solitary, immersed in the bark, erumpent through the surface of bark, causing a pustulate bark surface, with more than 10 perithecia arranged irregularly 1.9–2.8(–3.7) mm (av. = 2.5 ± 0.6 mm, *n* = 10) in diam. *Ectostromatic disc* orange, circular to oblong, with more than 10 ostioles arranged regularly to irregularly per disc (1–)1.1–2.2(–2.6) mm (av. = 1.7 ± 0.6 mm, *n* = 10) in diam. *Ostioles* numerous, scattered, umbilicate, sulcate, dark brown to black, at the same level as the disc (160–)170–200(–215) μm (av. = 184.6 ± 5.4 μm, *n* = 10) in diam. *Perithecia* outer surface lacking powdery entostroma, black, flask-shaped to spherical, with long discrete perithecial necks (525–)630–850(–970) μm (av. = 748.4 ± 119.4 μm, *n* = 10) in diam. *Asci* clavate to elongate obovoid, polysporous, thin-walled, long pedicellate, apically rounded to flat, 129–140 × (5–)8–12 μm (av. = 135 ± 4.5 × 9.8 ± 1.7 μm, *n* = 10). *Ascospores* elongate-allantoid, thin-walled, pale yellowish to pale brown at maturity, slightly curved, smooth, aseptate (4.5–)5–6.5(–7) × 1–1.5 μm (av. = 5.8 ± 0.6 × 1.3 ± 0.2 μm, *n* = 30). Asexual morph: not observed.

*Culture characteristics*: Cultures are white, dense, uniform, fluffy, growing up to 4 cm in diam. After 3 days, and reaching 9 cm within 10 days. Colonies do not produced pigmentation on PDA media.

*Known host and distribution*: Known only on *Betula albosinensis* in Hubei Province, China.

*Notes*: *Diatrypella shennongensis* can be distinguished from its closest relative, *Da. betulae*, by its number of perithecia (less than 10 vs. more than 10) in one stroma and base number difference (67/665 in ITS and 13/416 in *tub2*). In addition, the multigene phylogenetic analyses support this species as a new species with high statistical support (MP/ML/BI = 100/100/1).

***Eutypella*** (Nitschke) Sacc., Atti Soc. Veneto-Trent. Sci. Nat., Padua, Sér. 44: 80, 1875.

*Type*: *Eutypella cerviculata* (Fr.) Sacc., Syll. Fung. (Abellini) 1: 146, 1882.

*Known distribution*: Asia, Europe, North America, Oceania, South Africa, and South America (Doidge, 1950; Rappaz, 1987; Trouillas et al., 2011; Jayawardena et al., 2018).

*Notes*: The genus *Eutypella* was established by Saccardo (1875) with *E. cerviculata* as the type species (Saccardo, 1882). *Eutypella* species was mainly associated with canker diseases in *Vitis vinifera* (Vasilyeva and Stephenson, 2006; Trouillas et al., 2011; Luque et al., 2012). Although 251 species epithets of *Eutypella* have been listed in Index Fungorum (2021), the most of species are lacking DNA sequence. In phylogenetic analyses of *Diatrypaceae*, it showed that *Eutypella* was polyphyletic (Acero et al., 2004; Chacón et al., 2013; de Almeida et al., 2016; Shang et al., 2017). Thus, further studies of the taxa in *Eutypella* is needed.

***Eutypella citricola*** Speg., Anales del Museo Nacional de Buenos Aires 6: 245, 1898.

*Descriptions*: see Trouillas et al. (2011).

*Known host and distribution*: Known from *Citrus limon*, *C. sinensis*, *C. paradisi*, *Schinus molle* var. *areira*, *Ulmus procera* in Australia; *Vitis vinifera* in United States, *Morus alba* in China.

*Collections examined*: CHINA. Jiangsu Province, Yangzhou City, 119°28′11.81″ E, 32°47′25.10″ N, 1 m msl., from branches of *Morus alba*, 12 Nov. 2017, C.M. Tian & N. Jiang, BJFU CF2020520, living culture CFCC 52433; ibid., BJFU CF2020521, living culture CFCC 52434.

*Notes*: The current strains CFCC 52433 and CFCC 52434 cluster in a well-supported clade (MP/ML/BI = 100/100/1) with *Eutypella citricola* (HVVIT07 and HVGRF01), residing in *Allocryptovalsa*/*Eutypella sensu lato* (Figure 1; Clade 12). In morphology, this fungus can be identified by its 3–4 sulcate ostioles, 8-spored, 55–80 × 7.5–9 μm asci and (9–)10.5–12(–13) × 2–3 μm ascospores (Trouillas et al., 2011). This study represents the first record of this species from *Morus alba*. Species of *Allocryptovalsa* represents similarity to *Cryptovalsa* in morphology by having polysporous asci and is different from the eight spored asci of *Eutypella* (Senwanna et al., 2017). Unfortunately, we did not observe any microscopic feature from premature ascostromata. Thus, the current identification is preliminary and tentative, as it requires typification before a stable species concept achieved.

### Other Genera Included in *Diatrypaceae*

In here, we follow Hyde et al. (2020a); Wijayawardene et al. (2020) to list the genera in *Diatrypaceae*. A taxonomic key to distinguish 23 genera of *Diatrypaceae* is provided.

***Allodiatrype*** Konta & K.D. Hyde, Mycosphere 11(1): 247, 2020.

MycoBank MB 1814.

*Type*: *Allodiatrype arengae* Konta & K.D. Hyde, Mycosphere 11(1): 249, 2020.

*Known distribution*: Thailand (Li et al., 2016; Konta et al., 2020).

*Notes*: *Allodiatrype* was established by Konta et al. (2020) and typified by *A. arengae.* In the meanwhile, *A. elaeidicola*, *A. elaeidis*, and *A. thailandica* were also accommodated to this genus (Konta et al., 2020). The genus shares most similarities with *Diatrype*, whereas they can be distinguished by the shape and size of stromata (Konta et al., 2020).

***Anthostoma*** Nitschke, Pyrenomyc. Germ. 1: 110, 1867.

MycoBank MB 224.

*Type*: *Anthostoma decipiens* (DC.) Nitschke, Pyrenomyc. Germ. 1: 111, 1867.

*Known distribution*: Asia, Europe, North America, and South America (Cash, 1952; Ahmad, 1969; Farr, 1973; Rappaz, 1995).

*Notes*: Both sexual and asexual morphs of the type species *Anthostoma decipiens* were studied by Rappaz (1992, 1993). Phylogenetic analyses of sequence data also supported these results (MP/ML/BI = 100/100/1) (Rocchi et al., 2010; Jaklitsch et al., 2014). Species of *Anthostoma* own dark brown to dark, globose to subglobose ascomata, cylindrical, prominent ostioles, cylindrical to clavate asci with apically rounded to truncate apices and a short pedicel and brown to black-brown ascospores (Nitschke, 1867).

***Cryptosphaeria*** Ces. & De Not., Comm. Soc. Crittog. Ital. 1(4): 231, 1863.

MycoBank MB 26092.

*Type*: *Cryptosphaeria millepunctata* Grev., Fl. Edin.: 360, 1824.

*Known distribution*: Asia, Europe, North America, Oceania, and South Africa (Teodoro, 1937; Trouillas et al., 2015; Jayawardena et al., 2018; Moyo et al., 2019).

*Notes*: *Cryptosphaeria* is widely accepted in *Diatrypaceae* (Nitschke, 1867, 1870; Rappaz, 1989; Trouillas et al., 2010b, 2015). The genus comprises corticolous species and is characterized by widely effuse and poorly developed stromata, often covered by a periderm, with separately emerging ostioles, spindle-shaped, long pedicellate asci, and sub-olivaceous to brown ascospores (Glawe, 1984; Rappaz, 1987).

***Cryptovalsa*** Ces. & De Not. ex Fuckel, Jb. Nassau. Ver. Naturk. 23–24: 212, 1870.

MycoBank MB 1340.

*Type*: *Cryptovalsa protracta* (Pers.) De Not., Hedwigia 2: 178, 1863.

*Known distribution*: Asia, Europe, North America, Oceania, South Africa, and South America (Teodoro, 1937; Trouillas et al., 2010b, 2011; Jayawardena et al., 2018; Moyo et al., 2019).

*Notes*: *Cryptovalsa* was established by Cesati and De Notaris (1863) to accommodate *C*. *protracta*, *C*. *ampelina*, *C*. *nitschkei*, and *C*. *effusa*. The genus is characterized by eutypoid stromata that are rather variable, when erumpent separately diatrypelloid, often immersed in wood, but sometimes invading bark tissues. Asci are cylindrical or clavate, polysporous, with short or long pedicels. Ascospores are crowded, allantoid, and yellowish (Spooner, 1981; Vasilyeva and Stephenson, 2005). Fifty-five species epithets of *Cryptovalsa* are listed in Index Fungorum (2021), but only *Cryptovalsa ampelina* has sequence data.

***Diatrypasimilis*** J.J. Zhou & Kohlm., Mycologia 102(2): 432, 2010.

MycoBank MB 515026.

*Type*: *Diatrypasimilis australiensis* J.J. Zhou & Kohlm., Mycologia 102(2): 432, 2010.

*Known distribution*: Australia (Chalkley et al., 2010).

*Notes*: *Diatrypasimilis* was established to accommodate *D. australiensis* as type species from mangroves based on conventional taxonomic criteria and molecular phylogeny by Chalkley et al. (2010). The genus is characterized by carbonaceous, black stromata, 8-spored, cylindrical asci, and ellipsoidal, dark brown ascospores with a germ slit.

***Dothideovalsa*** Speg., Anal. Mus. Nac. B. Aires, Ser. 3(12): 414, 1909.

MycoBank MB 1697.

*Type*: *Dothideovalsa tucumanensis* Speg., Anal. Mus. Nac. B. Aires, Ser. 3(12): 414, 1909.

*Known distribution*: Argentina, Brazil, and United States (Rappaz, 1987; Hanlin, 1992).

*Notes*: *Dothideovalsa* was introduced to accommodate *D*. *diantherae*, *D*. *eutypoides*, *D*. *tucumanensis*, and *D*. *turnerae*. At present, only four species epithets of *Dothideovalsa* were listed in Index Fungorum (2021). However, there was no available sequence data in GenBank. Therefore, this genus is still doubtful and needs further studies.

***Endoxylina*** Romell, Bot. Notiser 1892: 173, 1892.

MycoBank MB 1814.

*Type*: *Endoxylina stellulata* Romell, Bot. Notiser: 173 (1892).

*Known distribution*: Asia, Europe, and North America (Tai, 1979; Ju et al., 1996).

*Notes*: *Endoxylina* was introduced and assigned to *Diatrypales* (Current name: *Xylariales fide* Kirk et al., 2008) without assigning the familial position by Romell (1892). Based on previous morphological literature and herbarium studies, Hyde et al. (2017) transferred *Endoxylina* to *Diatrypaceae*. The concept of genus *Endoxylina* is rather broad, and it is characterized as having stromata of valsoid or eutypoid configurations and 8-spored, long pedicellate, asci with J-, apical ring, as well as uni- to triseptate, ascospores (Romell, 1892; Ju et al., 1996; Vasilyeva, 2010; Hyde et al., 2017). At present, 21 species epithets of *Endoxylina* have been described in Index Fungorum (2021), however, some of these *Endoxylina* species have now been transferred and synonymized with other genera (Ellis and Everhart, 1892; Wehmeyer, 1975; Rappaz, 1987; Barr, 1993). Therefore, the number of species recognized for this genus is not well-established.

***Eutypa*** Tul. & C. Tul., Select. Fung. Carpol. (Paris) 2: 52, 1863.

MycoBank MB 1950.

*Type*: *Eutypa lata* (Pers.) Tul. & C. Tul., Select. Fung. Carpol. (Paris) 2: 56, 1863.

*Known distribution*: Asia, Europe, North America, Oceania, South Africa, and South America (Ahmad, 1978; Rappaz, 1987; Schmid-Heckel, 1988; Eriksson and Yue, 1998; Mulenko et al., 2008; Moyo et al., 2019).

*Notes*: Species of *Eutypa* are the causal agents of dieback of grapevine, apricots, and cherries (Trouillas and Gubler, 2004, 2010; Baumgartner et al., 2010; Camps et al., 2010; Blanco-Ulate et al., 2013; Camps et al., 2014). The genus is characterized by stromata which are irregular in shape, as confluent bumps, with conspicuous, scattered, roundish to prominent ostioles on the host surface. Asci are 8-spored, clavate, apically rounded to truncate, with indistinct apical rings and long pedicels. Ascospores are allantoid to ellipsoidal, aseptate, and pale yellowish.

***Halocryptosphaeria*** Dayar., Devadatha, V.V. Sarma & K.D. Hyde, Mycosphere 11(1): 136, 2020.

MycoBank MB 556800.

*Type*: *Halocryptosphaeria bathurstensis* (K.D. Hyde & Rappaz) Dayar. & K.D. Hyde, Mycosphere 11(1): 136, 2020.

*Known distribution*: India (Dayarathne et al., 2020a).

*Notes*: *Halocryptosphaeria* was established by Dayarathne et al. (2020a) to accommodate only one species, *H. bathurstensisspecies*. Although *Halocryptosphaeria* has some morphological similarities with *Cryptosphaeria*, they can be easily distinguished by phylogenetical analyses.

***Halocryptovalsa*** Dayar. & K.D. Hyde, Cryptog. Mycol. 41(3): 49, 2020.

MycoBank MB 824308.

*Type*: *Halocryptovalsa avicenniae* (Abdel-Wahab, Bahkali & E.B.G. Jones) Dayar. & K.D. Hyde, Cryptog. Mycol. 41(3): 50, 2020.

*Known distribution*: India, Saudi Arabia, and Thailand (Abdel-Wahab et al., 2017; Dayarathne et al., 2020a, b).

*Notes*: *Halocryptovalsa* was established by Dayarathne et al. (2020b) to accommodate species resembling *Cryptovalsa* from marine environments namely *Cryptovalsa avicenniae* and a new species *Halocryptovalsa salicorniae*. The genus is characterized by poorly developed stromata and poly-spored asci, with a J-, cylindrical, conspicuous apical or subapical ring. Ascospores are hyaline or yellow-brown to brown, allantoid, with small, fat globules at the end (Dayarathne et al., 2020b).

***Halodiatrype*** Dayar. & K.D. Hyde, Phytotaxa 7(5): 617, 2016.

MycoBank MB 552254.

*Type*: *Halodiatrype salinicola* Dayar. & K.D. Hyde, Mycosphere 7(5): 617, 2016.

*Known distribution*: Thailand (Dayarathne et al., 2016, 2020b).

*Notes*: *Halodiatrype* was established by Dayarathne et al. (2016) to accommodate *H*. *avicenniae* and *H*. *salinicola* isolated from mangroves. The characteristic of this genus is having ascomata lacking stromatal tissues, 8-spored, cylindrical to clavate, pedicellate asci, oblong to allantoid or sub-inequilateral, larger ascospores with septa, and libertella-like asexual morphs, which can easily identify *Halodiatrype* from other genera in *Diatrypaceae* (Dayarathne et al., 2016; 2020b).

***Leptoperidia*** Rappaz, Mycol. Helv. 2(3): 544, 1987.

MycoBank MB 25186.

*Type*: *Leptoperidia macropunctata* (Rehm) Rappaz, Mycol. Helv. 2(3): 545, 1987.

*Known distribution*: Congo, Mexico, Philippines (Ellis and Everhart, 1896; Rehm, 1913).

*Notes*: *Leptoperidia* was introduced to accommodate *L*. *applanata*, *L*. *asperrima*, *L*. *macropunctata*, and *L*. *trifida* (Rappaz, 1987). The genus is characterized by relatively small stroma, asci and ascospores, perithecia with very thin and slightly melanized walls (Rappaz, 1987). At present, only four species epithets of *Leptoperidia* are listed in Index Fungorum (2021). Sequence data are unavailable in GenBank.

***Libertella*** Desm., Annls Sci. Nat., Bot. 19: 275, 1830.

MycoBank MB 8769.

*Type*: *Libertella betulina* Desm., Annls Sci. Nat., Bot. 19: 276, 1830.

*Known distribution*: Asia, Europe, North America, Oceania, South Africa, and South America (Doidge, 1950; Conners, 1967; Ahmad, 1969; Farr, 1973; Sosnowski et al., 2007; Mulenko et al., 2008).

*Notes*: *Libertella* was introduced by Desmazières (1830) to accommodate *L. betulina*, *L*. *faginea*, and *L*. *rosae*. This genus was mostly reported as the asexual morph of *Diatrypella*, however, some species were reported as the asexual morph of *Eutypa*, *Eutypella*, *Diaporthe*, and *Polystigma* (Kirk et al., 2001). The genus is characterized by subcortical, erumpent and yellow to red acervula conidiomata, and branched conidiophores that produce hyaline, 1-celled, filiform conidia (Barnett and Hunter, 1972; Sutton, 1980; von Arx, 1981).

***Monosporascus*** Pollack & Uecker, Mycologia 66(2): 348, 1974.

MycoBank MB 3260.

*Type*: *Monosporascus cannonballus* Pollack & Uecker, Mycologia 66(2): 348, 1974.

*Known distribution*: Africa, Asia, Europe, North America, and South America (Pande, 2008; Sales et al., 2010; Chew-Madinaveitia et al., 2012; Salem et al., 2013; Aleandri et al., 2017).

*Notes*: *Monosporascus* was introduced by Pollack and Uecker (1974) with *M*. *cannonballus* as the type species. The genus is characterized by pyriform asci and the formation of one (rarely two) single large, sphaerical ascospores (Pollack and Uecker, 1974).

***Neoeutypella*** M. Raza, Q.J. Shang, Phook. & L. Cai, Fungal Diversity 95: 167, 2019.

MycoBank MB 555373.

*Type*: *Neoeutypella baoshanensis* M. Raza, Q.J. Shang, Phook. & L. Cai, Fungal Diversity 95: 168, 2019.

*Known distribution*: China and France (Phookamsak et al., 2019).

*Notes*: *Neoeutypella* was introduced by Phookamsak et al. (2019) to accommodate two fungal strains under the name *Eutypella caricae* and a new strain isolated from *Pinus armandii* (*Pinaceae*). *Neoeutypella* is characterized by carbonaceous stromata, erumpent through host epidermis, producing yellow pigments surrounding the stroma, 8-spored, spindle-shaped asci with long pedicellate, and overlapping 1–3-seriate, allantoid, aseptate, slightly or moderately curved ascospores, with a libertella-like asexual morph (Phookamsak et al., 2019).

***Pedumispora*** K.D. Hyde & E.B.G. Jones, Mycol. Res. 96(1): 78, 1992.

MycoBank MB 25433.

*Type*: *Pedumispora rhizophorae* K.D. Hyde & E.B.G. Jones, Mycol. Res. 96: 78, 1992.

*Known distribution*: Australia, India, and Thailand (Hyde and Jones, 1992; Pande, 2008; Dayarathne et al., 2020b).

*Notes*: *Pedumispora* was established by Hyde and Jones (1992) to accommodate a taxon from mangrove habitats. A phylogenetic study based on nuclear ITS and LSU regions showed that the taxonomic position of *Pedumispora* was in *Diatrypaceae* (Klaysuban et al., 2014). The genus is characterized by 8-spored, fusiform, pedicellate, unitunicate, apically truncate asci. Ascospores are filiform, mutli-septate, curved, longitudinally striate, with tapering poles, with one or both ends crook-like (Hyde and Jones, 1992).

***Peroneutypa*** Berl., Icon. Fung. 3: 80, 1902.

MycoBank MB 3834.

*Type*: *Peroneutypa bellula* (Desm.) Berl., Icon. Fung. 3: 81, 1902.

*Known distribution*: Asia, Europe, South Africa, and South America (Teodoro, 1937; Moyo et al., 2018a, Castilla-Cayuman et al., 2019; Moyo et al., 2019).

*Notes*: *Peroneutypa* was introduced by Berlese (1902) to accommodate *P. bellula*, *P*. *corniculata, and P*. *heteracantha* without designating the type species. Rappaz (1987) proposed *P. bellula* as the type species and synonymized *Peroneutypa* under *Eutypella*. However, Carmarán et al. (2006) resurrected *Peroneutypa* based on morphological characters and phylogenetic. The genus is described as valsoid stromata, with packed, long prominent necks, sessile to long pedicels, small asci with truncate apices, and allantoid ascospores (Carmarán et al., 2006, 2014; Senwanna et al., 2017; Shang et al., 2017).

***Quaternaria*** Tul. & C. Tul., Select. Fung. Carpol. 2: 104, 1863.

MycoBank MB 4632.

*Type*: *Quaternaria persoonii* Tul. & C. Tul., Select. Fung. Carpol. 2: 105, 1863.

*Known distribution*: Asia, Europe, and South America (Mujica and Vergara, 1945; Munk, 1957; Srinivasulu and Sathe, 1970).

*Notes*: *Quaternaria* was introduced by Tulasne and Tulasne (1863) and was typified by *Q. persoonii*. Clements and Shear (1931) lectotypified the illegitimate name *Q*. *quaternata* to *Q*. *persoonii* and considered *Quaternaria* as a synonym of *Eutypella* (Tulasne and Tulasne, 1863). Based on molecular phylogeny and the discussion of Gams (1994), *Quaternaria* was considered to be an independent genus by Acero et al. (2004). The genus is characterized by stromata was cryptosphaeroid in appearance and developed within the bark parenchyma.

***Rostronitschkia*** Fitzp., Mycologia 11(4): 165, 1919.

MycoBank MB 4793.

*Type*: *Rostronitschkia nervincola* Fitzp., Mycologia 11(4): 166, 1919.

*Known distribution*: Puerto Rico and Virgin Islands (Stevenson, 1975).

*Notes*: *Rostronitschkia* was introduced to accommodate the type species *R*. *nervincola*. At present, only the type species was listed in Index Fungorum (2021). However, there were no available sequence data and strains. Therefore, this genus is still doubtful and needs to further study.

Key to genera of *Diatrypaceae*

1 Sexual morph absent………………………………………………………. 2
1 Sexual morph present…………………………………………………….. 3

2 Conidiomata acervuli, yellow to red………………….. *Libertella*
2 Conidiomata pycnidial, brownish yellow, watery, bubble-like…………………………………………………………………….. *Diatrype*

3 Ascospores globose, fusiform, or oblong to ellipsoidal…… 4
3 Ascospores allantoid……………………………………………………… 7

4 Ascospores fusiform, septate………………………. *Pedumispora*
4 Ascospores aseptate……………………………………………………….. 5

5 Ascospores globose, with 1–2 spores in each ascus………… ………………………………………………………………… *Monosporascus*
5 Ascospores oblong to ellipsoidal, with 8 spores in each ascus……………………………………………………………………………… 6

6 Ascospores with a germ slit……………………… *Diatrypasimilis*
6 Ascospores lacking a germ slit………………………. *Anthostoma*

7 Asci with more than 8 spores………………………………………… 8
7 Asci with 8 spores………………………………………………………….. 9

8 Stromata erumpent through host surface, discoid……. ……………………………………………………………………… *Diatrypella*
8 Stromata immersed in wood but sometimes invading bark tissues, eutypoid…………………………………………………………… 10

9 Perithecia outer surface lacking powdery entostroma………. ………………………………………………………………… *Allocryptovalsa*
9 Perithecia outer surface coated with white, powdery entostroma……………………………………………………. *Cryptovalsa*

10 Habitats marine…………………………………….. *Halocryptovalsa*
10 Habitats terrestrial……………………………………………………….. 11

11 Ascospores 0-1 septate…………………………………………………. 12
11 Ascospores uni- to triseptate………………………….. *Endoxylina*

12 Stromata semi-immersed to erumpent through the host periderm (ectostromatic)…………………………………………….. 13
12 Stromata deeply immersed in the host periderm (entostromatic)…………………………………………………………….. 15

13 Asci lacking an apical ring…………………………… *Halodiatrype*
13 Asci with J-, cylindrical, conspicuous apical ring…………. 14

14 Ascospores hyaline becoming yellowish at maturity…….. ……………………………………………………………………… *Allodiatrype*
14 Ascospores hyaline…………………………………………….. *Diatrype*

15 Ascomata clustered, forming valsoid configuration, breaking through entostroma by short to long necks….. 16
15 Ascomata scattered, arranged in linear entostroma, with short to long necks………………………………………………………. 18

16 Entostromata immersed in the host, with individually protruding necks at the center…………………….. *Quaternaria*
16 Entostromata slightly raised on the host, with long cylindrical, packed necks……………………………………………. 17

17 Hosts range wide……………………………………………. *Eutypella*
17 Host *Pinus armandii*…………………………………… *Neoeutypella*

18 Ascomata forming very long necks, through the host surface………………………………………………………… *Peroneutypa*
18 Ascomata forming short papilla protruding the host surface……………………………………………………………………….. 19

19 Peridium thin-walled, composed of a single layer of melanized cells, difficult to separate from the entostroma………………………………………………… *Leptoperidia*
19 Peridium thick-walled, composed of not one distinct layers, separating from entostroma………………………………………… 20

20 Asci cylindric-clavate, with pale yellow ascospores… *Eutypa*
20 Asci generally spindle-shaped, with sub-olivaceous to brown ascospores………………………………………………………… 21

21 Peridium compose of two distinct layers… *Cryptosphaeria*
21 Peridium compose of three distinct layers…………………… ………………………………………………………….. *Halocryptosphaeria*

Key to *Diatrypaceae* species on *Betula* spp.

1 Sexual morph absent……………………………… *Diatrype betulae*
1 Sexual morph present………………………………………………….. 2

2 Asci containing 8 biseriate ascospores…………………………… 3
2 Asci polysporous…………………………………………………………… 5

3 Apical ring amyloid…………………………….. *Diatrype undulata*
3 Apical ring indistinguishable………………………………………….. 4

4 Size of asci more than 25 μm………. *Cryptosphaeria venusta*
4 Size of asci less than 25 μm………………. *Diatrype platystoma*

5 Stromata immersed in wood but sometimes invading bark tissues, eutypoid……………………………….. *Eutypella halseyana*
5 Stromata erumpent through host surface, discoid………….. 6

6 Asci cylindrical………………………………… *Diatrypella favacea*
6 Asci clavate to spindle shaped………………………………………… 7

7 More than 10 perithecia arranged irregularly………. ……………………………………………….. *Diatrypella shennongensis*
7 Less than 10 perithecia arranged regularly……………………. 8

8 More than one host………………………… *Diatrypella betulicola*
8 Only one host…………………………………………………………………. 9

9 Length of asci less than 140 μm……………………… *Diatrypella betulae*
9 Length of asci more than 140 μm………………………………. ……………………………………………………… *Diatrypella hubeiensis*

## Discussion

In this study, 21 isolates were identified as diatrypaceous fungi from *Betula albosinensis*, *B. davurica*, and *B. platyphylla* (*Betulaceae*), *Castanea mollissima* and *Quercus mongolica* (*Fagaceae*), *Juglans regia* (*Juglandaceae*), and *Morus alba* (*Moraceae*). Morpho-molecular analyses confirmed that these strains belong in four genera (*viz*. *Allocryptovalsa*, *Diatrype*, *Diatrypella*, and *Eutypella*) including nine novel species (*viz*. *Allocryptovalsa castaneae*, *A. castaneicola*, *Diatrype betulae*, *D. castaneicola*, *D. quercicola*, *Diatrypella betulae*, *Da. betulicola*, *Da. hubeiensis*, and *Da. shennongensis*) and two known species (*viz*. *Diatrypella favacea* and *Eutypella citricola*). *Eutypella citricola* was reported from *Morus* host for the first time.

The generic concepts of *Diatrypaceae* have been unstable, thus many species were transferred from one genus to another (Phookamsak et al., 2019; Konta et al., 2020). The current study revised the *Diatrypaceae* and accepted 23 genera in this family (Supplementary Table 1). However, there only exist 18 genera in current phylogenetic analyses due to availability of molecular data (Figure 1). In China, 11 genera and 62 species belong in *Diatrypaceae* have been recorded (Supplementary Table 5). However, 40 species (66.67%) do not have molecular data until now.

Birch is of high economic, medicinal, and ornamental value. Six species of *Diatrypaceae* were recorded from *Betula* spp. with DNA sequences in the current study, including *Diatrype betulae*, *Diatrypella betulae*, *Da. betulicola*, *Da. favacea*, *Da. hubeiensis*, and *Da. shennongensis*. All species of *Diatrypella* 2 clade were isolated from *Betula* spp., except for *Da. pulvinata* and *Da. yunnanensis* isolated from an unidentified plant. It showed that many *Diatrypella* species may have obvious host specificity. The other four species (*viz*. *Cryptosphaeria venusta*, *Diatrype platystoma*, *D. undulata*, and *Eutypella halseyana*) of *Diatrypaceae* were recorded from *Betula* spp. in China but no materials with DNA sequences, including *Cryptosphaeria venusta*, *Diatrype platystoma*, *D. undulata*, and *Eutypella halseyana* (Teng, 1996; Vasilyeva and Ma, 2014). A morphological key was provided to separate them in the current study.

The *Allocryptovalsa* species clustered within the same clade as *Eutypella* species in our phylogenetic analyses (Figure 1; Clade 12: *Allocryptovalsa*/*Eutypella sensu lato*). *Allocryptovalsa* was introduced by Senwanna et al. (2017) which resembles *Cryptovalsa* in morphology by having polysporous asci different from the 8-spored asci of *Eutypella*. The number of ascospores per ascus (eight spores vs. multiple spores) has been used traditionally to differentiate the genera of *Diatrypaceae* (*Diatrype* vs. *Diatrypella* and *Cryptovalsa* vs. *Eutypella*). However, the recent studies indicated that the polysporous ascus feature maybe not significant in *Diatrypaceae* (Acero et al., 2004; Trouillas et al., 2011; Chacón et al., 2013; Liu et al., 2015). A thorough revision is needed to resolve the problematic situation of *Diatrypaceae*, which includes a mass of misidentified genus/species, as a result of the unstable phylogenetic frame with type materials. Therefore, it seems to better if the future work could treate the strains from clade 12 into one genus *Allocryptovalsa*.

The strains of *Diatrype* formed a clade with high support values (Figure 1; Clade 09: *Diatrype sensu stricto*). However, in this clade, some strains of *Diatrypella* (*Da. quercina*, *Da. iranensis*, and *Da. macrospora*) were placed between *Diatrype* species. Acero et al. (2004) reported that *Cryptosphaeria*, *Diatrype*, *Diatrypella*, *Eutypa*, and *Eutypella* were polyphyletic and confused probably due to lack of *tub2* gene sequences or misidentified species. However, these five genera are still polyphyletic within the family from previous studies (de Almeida et al., 2016; Shang et al., 2017; Mehrabi et al., 2019; Dayarathne et al., 2020a, b; Konta et al., 2020) based on the ITS and *tub2* sequences data. *Allodiatrype* and *Halodiatrype* reported their morphological resemblance to *Diatrype* (Dayarathne et al., 2016; Konta et al., 2020). However, asci of *Halodiatrype* lack an apical ring, the asci of *Allodiatrype* and *Diatrype* have J-, cylindrical, conspicuous apical ring (Dayarathne et al., 2016; Maharachchikumbura et al., 2016; Konta et al., 2020). Additionally, *Diatrype* can be differentiated from *Allodiatrype* by the color of ascospores. The ascospores of *Diatrype* are hyaline becoming yellowish ascospores at maturity, whereas the ascospores of *Allodiatrype* are hyaline (Maharachchikumbura et al., 2016; Konta et al., 2020). Though *Diatrype* and *Diatrypella* are not polyphyletic any more, some strains of *Diatrype* and *Diatrypella* species still need further study.

In some cases, some strains of *Diatrype* species (*D. brunneospora* and *D. palmicola*), *Diatrypella* species (*Da. banksiae*), *Eutypa* species (*E. flavovirens* and *E. guttulata*) and *Eutypalla* species (*E. cearensis*) formed distinct lineages within *Diatrypaceae* (Figure 1; *Incertae sedis*). *Diatrypella banksiae* is closely related to the genus *Neoeutypella* with high support values from the phylogenetic analyses (MP/ML/BI = 98/100/1) (Figure 1), which produced an asexual morph (Crous et al., 2016; Phookamsak et al., 2019). Nevertheless, *Diatrypella banksiae* having spindle-shaped conidia can be easily differentiated from *Neoeutypella baoshanensis* having filiform conidia (Crous et al., 2016; Phookamsak et al., 2019). In the future study, sexual morph of *Diatrypella banksiae* also remains to be studied, because the asexual morphs of *Diatrypaceae* are not generally useful in separating species (Glawe and Rogers, 1982, 1984; Rappaz, 1987; Konta et al., 2020). Therefore, *Diatrypella banksiae* probably belongs to the genus *Neoeutypella*. Further study of the relationship between *Neoeutypella* and *Diatrypella banksiae* is needed.

*Eutypa guttulata* is closely related to the genus *Halodiatrype* and *Pedumispora* from the phylogenetic analyses (Figure 1) as a basal branch. *Eutypa guttulata* can be distinguished by fusiform ascospores, whereas *Pedumispora* has allantoid ascospores (Hyde and Jones, 1992). It is also different from *Halodiatrype* because of lacking an apical ring (Dayarathne et al., 2016). *Diatrype brunneospora* is closed to *Eutypa guttulata* (Figure 1), which is morphologically similar to members of *Eutypa* spp. (Trouillas et al., 2010b). Therefore, the assignment of this isolate to the genus *Diatrype* may require reconsideration in the future. *Eutypa flavovirens* is closely related to the genus *Cryptosphaeria* with no support (Figure 1). *Diatrypella* can be distinguished by hyaline to subhyaline, rarely pale olivaceous ascospores, whereas *Diatrype palmicola* has pale yellowish to pale brown ascospores at maturity (Liu et al., 2015). For those species, the assignment of these isolates still remain unclear, which may require reconsideration in the future.

*Eutypa microasca* appeared in a strongly supported clade along with two *Peroneutypa* species with fusiform asci in Figure 1 (Clade 19: *Peroneutypa*). Carmarán et al. (2006) suggested that the morphology of the ascus could explain the phylogenetic relationships within *Diatrypaceae* better than stromata, although our study indicates that it is not entirely supported in *Peroneutypa*. The phylogenetic signal of the ascus shape and the phylogenetic placement of *Eutypa microasca* should be further tested in future study.

## Data Availability Statement

The datasets presented in this study can be found in online repositories. The names of the repository/repositories and accession number(s) can be found in the article/Supplementary Material.

## Author Contributions

XF and CT conceived and designed the experiments. HZ, MP, and RM performed the experiment. HZ and MP analyzed the data. NW and DD provided some materials and polished the language. HZ wrote the manuscript. XF revised and approved the final version of the manuscript. All authors contributed extensively to the work presented in the manuscript.

## Conflict of Interest

The authors declare that the research was conducted in the absence of any commercial or financial relationships that could be construed as a potential conflict of interest.

## Footnotes

1 www.mycobank.org

2 www.treebase.org

3 https://nt.ars-grin.gov/fungaldatabases

## Supplementary Material

Supplementary Table 1 Placement of genera in *Diatrypaceae* by different authors.

Supplementary Table 2 Isolates and GenBank accession numbers used in the phylogenetic analyses of *Diatrypaceae*.

Supplementary Table 3 Synopsis of species of *Allocryptovalsa*.

Supplementary Table 4 Synopsis of species of *Diatrypella* from *Betula* spp.

Supplementary Table 5 Distribution of diatrypaceous fungi on plants in China.
