# Supplementary material for: The Hidden Diversity of Diatrypaceous Fungi in China
Source: Front Microbiol. 2021 May 31;12:646262. doi: 10.3389/fmicb.2021.646262 (PMC8200573; doi:10.3389/fmicb.2021.646262)
Supplement: Supplementary Table 1 — Placement of genera in Diatrypaceae by different authors. [file Table_1.DOCX]

**Table S1.** Placement of genera in *Diatrypaceae* by different authors.

| Nitschke (1867)  (5 gen.) | Kirk et al. (2001)  (9 gen.) | Kirk et al. (2008) (13 gen.) | Senanayake et al. (2015) (17 gen.) | Wijayawardene et al. (2018) (17 gen.) | Wijayawardene et al. (2020) Hyde et al. (2020a) (20 gen.) | This study  (23 gen.) |
| --- | --- | --- | --- | --- | --- | --- |
| *Calosphaeria* | *Cryptosphaeria* | *Cryptosphaeria* | *Anthostoma* | *Allocryptovalsa* | *Allocryptovalsa* | *Allocryptovalsa* |
| *Diatrype* | *Cryptovalsa* | *Diatrype* | *Cryptosphaeria* | *Anthostoma* | *Anthostoma* | *Allodiatrype* |
| *Diatrypella* | *Diatrype* | *Dothideovalsa* | *Cryptovalsa* | *Cryptosphaeria* | *Cryptosphaeria* | *Anthostoma* |
| *Quaternaria* | *Diatrypella* | *Diatrypella* | *Diatrype* | *Cryptovalsa* | *Cryptovalsa* | *Cryptosphaeria* |
| *Scoptria* | *Eutypella* | *Eutypa* | *Diatrypella* | *Diatrypasimilis* | *Diatrypasimilis* | *Cryptovalsa* |
|  | *Echinomyces* | *Echinomyces* | *Diamantinia* | *Diatrype* | *Diatrype* | *Diatrypasimilis* |
|  | *Eutypa* | *Endoxylina* | *Diatrypasimilis* | *Diatrypella* | *Diatrypella* | *Diatrype* |
|  | *Fassia* | *Eutypella* | *Echinomyces* | *Echinomyces* | *Echinomyces* | *Diatrypella* |
|  | *Leptoperidia* | *Leptoperidia* | *Eutypa* | *Endoxylina* | *Endoxylina* | *Dothideovalsa** |
|  |  | *Libertella* | *Eutypella* | *Eutypa* | *Eutypa* | *Endoxylina** |
|  |  | *Quaternaria* | *Leptoperidia* | *Eutypella* | *Eutypella* | *Eutypa* |
|  |  | *Peroneutypa* | *Libertella* | *Leptoperidia* | *Halocryptovalsa* | *Eutypella* |
|  |  | *Rostronitschkia* | *Monosporascus* | *Halodiatrype* | *Halodiatrype* | *Halocryptosphaeria* |
|  |  |  | *Pedumispora* | *Libertella* | *Leptoperidia* | *Halocryptovalsa* |
|  |  |  | *Peroneutypa* | *Monosporascus* | *Libertella* | *Halodiatrype* |
|  |  |  | *Phaeoisaria* | *Peroneutypa* | *Monosporascus* | *Leptoperidia** |
|  |  |  | *Quaternaria* | *Quaternaria* | *Neoeutypella* | *Libertella** |
|  |  |  |  |  | *Pedumispora* | *Monosporascus* |
|  |  |  |  |  | *Peroneutypa* | *Neoeutypella* |
|  |  |  |  |  | *Quaternaria* | *Pedumispora* |
|  |  |  |  |  |  | *Peroneutypa* |
|  |  |  |  |  |  | *Quaternaria* |
|  |  |  |  |  |  | *Rostronitschkia** |

Notes: Genera without available sequence data in this study are marked by an asterisk (*).
