# Supplementary material for: The Hidden Diversity of Diatrypaceous Fungi in China
Source: Front Microbiol. 2021 May 31;12:646262. doi: 10.3389/fmicb.2021.646262 (PMC8200573; doi:10.3389/fmicb.2021.646262)
Supplement: Supplementary Table 2 — Isolates and GenBank accession numbers used in the phylogenetic analyses of Diatrypaceae. [file Table_2.DOCX]

**Table S2.** Isolates and GenBank accession numbers used in the phylogenetic analyses of *Diatrypaceae*.

| **Species** | **Strain** | **Host/Substrate** | **Origin** | **GenBank accession numbers** | |
| --- | --- | --- | --- | --- | --- |
|  |  |  |  | **ITS** | **TUB2** |
| ***Allocryptovalsa castanea*** | **CFCC 52428*** | *Castanea mollissima* | China | MW632945 | MW656393 |
| *Allocryptovalsa castanea* | CFCC 52427* | *Juglans regia* | China | MW632944 | MW656392 |
| *Allocryptovalsa castanea* | CFCC 52429* | *Castanea mollissima* | China | MW632946 | MW656394 |
| ***Allocryptovalsa castaneicola*** | **CFCC 52432*** | *Castanea mollissima* | China | MW632947 | MW656395 |
| ***Allocryptovalsa cryptovalsoidea*** | **HVFIG02** | *Ficus carica* | Australia | HQ692573 | HQ692524 |
| *Allocryptovalsa cryptovalsoidea* | HVFIG05 | *Ficus carica* | Australia | HQ692574 | HQ692525 |
| ***Allocryptovalsa elaeidis*** | **MFLUCC 15-0707** | *Elaeis guineensis* | Thailand | MN308410 | MN340296 |
| ***Allocryptovalsa polyspora*^TS^** | **MFLUCC 17-0364** | *Hevea brasiliensis* | Thailand | MF959500 | NA |
| *Allocryptovalsa rabenhorstii* | WA07CO | *Vitis vinifera* | Australia | HQ692620 | HQ692522 |
| *Allocryptovalsa rabenhorstii* | WA08CB | *Vitis vinifera* | Australia | HQ692619 | HQ692523 |
| ***Allocryptovalsa truncata*** | **NFCCI-4520** | NA | Inidia | MK990279 | NA |
| ***Allodiatrype arengae*^TS^** | **MFLUCC 15-0713** | *Arenga pinnata* | Thailand | MN308411 | MN340297 |
| ***Allodiatrype elaeidicola*** | **MFLUCC 15-0737a** | *Elaeis guineensis* | Thailand | MN308415 | MN340299 |
| ***Allodiatrype elaeidicola*** | **MFLUCC 15-0737b** | *Elaeis guineensis* | Thailand | MN308416 | NA |
| ***Allodiatrype elaeidis*** | **MFLUCC 15-0708a** | *Elaeis guineensis* | Thailand | MN308412 | MN340298 |
| ***Allodiatrype elaeidis*** | **MFLUCC 15-0708b** | *Elaeis guineensis* | Thailand | MN308413 | NA |
| *Allodiatrype thailandica* | MFLUCC 15-0711 | *Calamus* sp. | Thailand | MN308414 | NA |
| ***Allodiatrype thailandica*** | **MFLUCC 14-1210** | NA | Thailand | KU315392 | NA |
| *Anthostoma decipiens*^TS^ | IPV-FW349 | NA | Italy | AM399021 | NA |
| *Anthostoma decipiens*^TS^ | JL567 | *Vitis vinifera* | Spain | JN975370 | JN975407 |
| *Cryptosphaeria eunomia* var. *eunomia* | CBS 216.87 | *Fraxinus excelsior* | Switzerland | AJ302417 | NA |
| *Cryptosphaeria eunomia* var. *fraxini* | CBS 223.87 | *Fraxinus excelsior* | Switzerland | AJ302421 | KT425166 |
| ***Cryptosphaeria ligniota*** | **CBS 273.87** | *Populus tremula* | Switzerland | KT425233 | KT425168 |
| ***Cryptosphaeria multicontinentalis*** | **CBS 132918** | *Populus balsamifera* subsp. *trichocarpa* | Australia | KT425237 | KT425172 |
| ***Cryptosphaeria pullmanensis*** | **ATCC 52655** | *Populus trichocarpa* | USA | KT425235 | KT425170 |
| ***Cryptosphaeria subcutanea*** | **CBS 240.87** | NA | Norway | KT425232 | KT425167 |
| *Cryptosphaeria subcutanea* | DSUB100A | NA | Norway | KT425189 | KT425124 |
| *Cryptovalsa ampelina* | A001 | NA | Australia | GQ293901 | GQ293972 |
| *Cryptovalsa ampelina* | DRO101 | NA | USA | GQ293902 | GQ293982 |
| ***Diatrypasimilis australiensis*^TS^** | **ATCC MYA-3540** | *Rhizophora* sp. | Australia | FJ430590 | NA |
| ***Diatrype betulae*** | **CFCC 52416*** | *Betula davurica* | China | MW632943 | MW656391 |
| ***Diatrype brunneospora*** | **CNP01** | *Acacia longifolia* subsp. *sophorae* | Australia | HM581946 | HQ692478 |
| *Diatrype bullata* | UCDDCh400 | NA | USA | DQ006946 | DQ007002 |
| ***Diatrype castaneicola*** | **CFCC 52425*** | *Castanea mollissima* | China | MW632941 | MW656389 |
| *Diatrype castaneicola* | CFCC 52426* | *Castanea mollissima* | China | MW632942 | MW656390 |
| *Diatrype disciformis*^TS^ | CBS 205.87 | *Fagus sylvatica* | Switzerland | AJ302437 | NA |
| *Diatrype disciformis*^TS^ | GB 5815 | *Fagus grandifolia* | USA | AJ302423 | NA |
| ***Diatrype iranensis***  **(*Diatrypella iranensis*)** | **IRAN 2280C** | *Quercus brantii* | Iran | KM245033 | NA |
| ***Diatrype macrospora***  **(*Diatrypella macrospora*)** | **IRAN 2344C** | *Quercus brantii* | Iran | KR605648 | NA |
| ***Diatrype palmicola*** | **MFLUCC 11-0018** | *Caryota urens* | Thailand | KP744438 | NA |
| *Diatrype palmicola* | MFLUCC 11-0020 | *Caryota urens* | Thailand | KP744439 | NA |
| ***Diatrype quercicola*** | **CFCC 52418*** | *Quercus mongolica* | China | MW632938 | MW656386 |
| *Diatrype quercicola* | CFCC 52419* | *Quercus mongolica* | China | MW632939 | MW656387 |
| *Diatrype quercicola* | CFCC 52420* | *Quercus mongolica* | China | MW632940 | MW656388 |
| *Diatrype spilomea* | CBS 212.87 | *Acer campestre* | Switzerland | AJ302433 | NA |
| *Diatrype stigma* | DCASH200 | *Quercus* sp. | USA | GQ293947 | GQ294003 |
| *Diatrype stigma* | UCDDCash200 | NA | NA | DQ006945 | DQ007003 |
| *Diatrype undulata* | CBS 271.87 | *Betula* sp. | Switzerland | AJ302436 | NA |
| *Diatrype virescens* | CBS 128344 | NA | NA | MH864890 | NA |
| *Diatrypella atlantica* | HUEFS 136873 | unidentified plant | Brazil | KM396614 | KR259647 |
| ***Diatrypella atlantica*** | **HUEFS 194228** | unidentified plant | Brazil | KM396615 | KR363998 |
| *Diatrypella banksiae* | CPC 29054 | *Banksia coccinia* | Australia | KY173401 | NA |
| ***Diatrypella banksiae*** | **CPC 29118** | *Banksia formosa* | Australia | KY173402 | NA |
| ***Diatrypella betulae*** | **CFCC 52406*** | *Betula albosinensis* | China | MW632931 | MW656379 |
| *Diatrypella betulae* | CFCC 52404* | *Betula albosinensis* | China | MW632929 | MW656377 |
| *Diatrypella betulae* | CFCC 52405* | *Betula albosinensis* | China | MW632930 | MW656378 |
| ***Diatrypella betulicola*** | **CFCC 52411*** | *Betula davurica* | China | MW632935 | MW656383 |
| *Diatrypella betulicola* | CFCC 52412* | *Betula platyphylla* | China | MW632936 | MW656384 |
| *Diatrypella delonicis* | MFLU 16-1032 | *Delonix regia* | Thailand | MH812995 | MH847791 |
| *Diatrypella delonicis* | MFLUCC 15-1014 | *Delonix regia* | Thailand | MH812994 | MH847790 |
| ***Diatrypella elaeidis*** | **MFLUCC 15-0279** | *Elaeis guineensis* | Thailand | MN308417 | MN340300 |
| *Diatrypella favacea*^TSQ^ | CBS 198.49 | *Betula pendula* | NA | MH856491 | NA |
| ***Diatrypella favacea*^TSQ^** | **CFCC 52409*** | *Betula platyphylla* | China | MW632934 | MW656382 |
| *Diatrypella favacea*^TSQ^ | DL26C | *Betula* sp. | Netherlands | AJ302440 | NA |
| *Diatrypella favacea*^TSQ^ | R191 | *Betula* sp. | Lithuania | JN689955 | NA |
| *Diatrypella frostii* | UFMGCB 1917 | *Solanum cernuum* | Brazil | HQ377280 | NA |
| ***Diatrypella heveae*** | **MFLUCC 17-0368** | *Hevea brasiliensis* | Thailand | MF959501 | MG334557 |
| ***Diatrypella hubeiensis*** | **CFCC 52413*** | *Betula davurica* | China | MW632937 | MW656385 |
| *Diatrypella major* | ANM 1947 | NA | USA | KU320613 | NA |
| *Diatrypella prominens* | ATCC 64182 | *Plantanus* sp. | USA | AJ302442 | NA |
| *Diatrypella pulvinata* | DL29C | NA | NA | AJ302443 | NA |
| *Diatrypella pulvinata* | H048 | *Salix alba* | Czech Republic | FR715523 | FR715495 |
| *Diatrype quercina*  (*Diatrypella quercina*) | F-091966 | *Quercus faginea* | Spain | AJ302444 | NA |
| *Diatrypella shennongensis* | CFCC 52414* | *Betula albosinensis* | China | MW632932 | MW656380 |
| ***Diatrypella shennongensis*** | **CFCC 52415*** | *Betula albosinensis* | China | MW632933 | MW656381 |
| ***Diatrypella tectonae*** | **MFLUCC 12-0172a** | *Tectona grandis* | Thailand | KY283084 | NA |
| ***Diatrypella tectonae*** | **MFLUCC 12-0172b** | *Tectona grandis* | Thailand | KY283085 | NA |
| *Diatrypella verruciformis*^TSQ^ | UCROK1467 | *Quercus agrifolia* | USA | JX144793 | JX174093 |
| *Diatrypella verruciformis*^TSQ^ | UCROK754 | *Quercus agrifolia* | USA | JX144783 | JX174083 |
| *Diatrypella vulgaris* | HVFRA02 | *Fraxinus angustifolia* | Australia | HQ692591 | HQ692503 |
| ***[Diatrypella vulgaris](https://www.ncbi.nlm.nih.gov/nuccore/MH876328.1)*** | **HVGRF03** | *Citrus paradisi* | Australia | HQ692590 | HQ692502 |
| ***Diatrypella yunnanensis*** | **JZBH3380001** | unidentified plant | China | MN653008 | MN887112 |
| *Eutypa astroidea* | CBS 292.87 | NA | Switzerland | AJ302458 | DQ006966 |
| *Eutypa consobrina* | STEU 8153 | NA | South Africa | MF359628 | MF359661 |
| ***Eutypa cremea*** | **STEU 8082** | *Vitis vinifera* | South Africa | KY111656 | KY111598 |
| *Eutypa cremea* | STEU 8410 | *Prunus armeniaca* | South Africa | KY752765 | KY752789 |
| *Eutypa crustata* | CBS 210.87 | *Ulmus* sp. | France | AJ302448 | DQ006968 |
| *Eutypa flavovirens* | CBS 272.87 | *Quercus ilex* | France | AJ302457 | DQ006959 |
| ***Eutypa guttulata*** | **HUEFS 192075** | unidentified plant | Brazil | KM396637 | NA |
| *Eutypa laevata* | CBS 291.87 | *Salix* sp. | Switzerland | HM164737 | HM164771 |
| *Eutypa lata*^TS^ | EP18 | *Vitis vinifera* | New South Wales | HQ692611 | HQ692501 |
| *Eutypa lata*^TS^ | JL399 | Cabernet Sauvignon | Spain | JN975339 | JN975376 |
| *Eutypa lata* var. *aceris* | CBS 217.87 | *Acer campestre* | France | HM164734 | HM164768 |
| *Eutypa lata*^TS^ (*Eutypa armeniacae*) | ATCC 28120 | NA | Australia | DQ006948 | DQ006975 |
| *Eutypa lata*^TS^ (*Eutypa armeniacae*) | CBS 622.84 | *Vitis vinifera* | Italy | AJ302446 | DQ006964 |
| *Eutypa lejoplaca* | 020202-3 | *Acer pseudoplatanus* | Switzerland | AY684238 | AY684197 |
| *Eutypa lejoplaca* | 020202-5 | *Acer pseudoplatanus* | Switzerland | AY684221 | AY684196 |
| *Eutypa leptoplaca* | CBS 287.87 | *Frangula alnus* | Switzerland | AY684226 | AY684204 |
| *Eutypa leptoplaca* | CBS 288.87 | *Cyssus hypoglauca* | Australia | AY684227 | AY684205 |
| *Eutypa maura* | CBS 219.87 | *Vitis vinifera* | Switzerland | DQ006926 | DQ006967 |
| *Eutypa petrakii* var. *hederae* | CBS 285.87 | NA | Switzerland | MH862077 | NA |
| *Eutypa petrakii* var*. petrakii* | CBS 245.87 | NA | Norway | AJ302456 | DQ006971 |
| *Eutypa sparsa* | 3802-3a | *Populus* sp. | Switzerland | AY684219 | AY684200 |
| *Eutypa sparsa* | 3802-3b | *Populus* sp. | Switzerland | AY684220 | AY684201 |
| *Eutypa tetragona* | CBS 284.87 | *Sarothamnus scoparius* | France | DQ006923 | DQ006960 |
| ***Eutypella australiensis*** | **CNP03** | *Acacia longifolia* subsp. *sophorae* | Australia | HM581945 | HQ692479 |
| ***Eutypella cearensis*** | **HUEFS 131070** | Unidentified plant | Brazil | KM396639 | NA |
| *Eutypella cerviculata* | CBS 221.87 | *Alnus glutinosa* | Switzerland | AJ302468 | NA |
| *Eutypella cerviculata* | M68 | *Alnus glutinosa* | Latvia | JF340269 | NA |
| *Eutypella citricola* | HVGRF01 | *Citrus sinensis* | Australia | HQ692589 | HQ692521 |
| *Eutypella citricola* | HVVIT07 | *Vitis vinifera* | Australia | HQ692579 | HQ692512 |
| *Eutypella citricola* | CFCC 52433* | *Morus alba* | China | MW632948 | MW656396 |
| *Eutypella citricola* | CFCC 52434* | *Morus alba* | China | MW632949 | MW656397 |
| *Eutypella leprosa* | STEU 8189 | NA | South Africa | MF359637 | MF359672 |
| *Eutypella leprosa* | STEU 8190 | NA | South Africa | MF359638 | MF359673 |
| *Eutypella microtheca* | ADEL200 | *Ulmus procera* | Australia | HQ692559 | HQ692527 |
| *Eutypella microtheca* | BCMX01 | *Vitis vinifera* | Mexico | KC405563 | KC405560 |
| *Eutypella microtheca* | CBS 128337 | *Citrus paradisi* | Australia | MH864886 | NA |
| ***Eutypella persica*** | **IRAN 2540C** | *Alnus* sp. | Iran | KX828144 | NA |
| ***Eutypella quercina*** | **IRAN 2543C** | *Quercus* sp*.* | Iran | KX828139 | NA |
| ***Eutypella semicircularis*** | **MP4669** | *Alnus acuminata* | Panama | JQ517314 | NA |
| *Eutypella vitis* | UCD2291AR | *Vitis vinifera* | USA | HQ288224 | HQ288303 |
| *Eutypella vitis* | UCD2428TX | *Vitis vinifera* | Texas, USA | FJ790851 | GU294726 |
| ***Halocryptosphaeria bathurstensis*^TS^** | **NFCCI-4248** | *Avicennia marina* | India | MN061366 | MN431496 |
| ***Halocryptovalsa salicorniae*^TS^** | **MFLUCC 15-0185** | *Salicornia sp.* | Thailand | MH304410 | MH370274 |
| ***Halodiatrype avicenniae*** | **MFLUCC 15-0953** | *Avicennia* sp. | Thailand | KX573916 | KX573931 |
| ***Halodiatrype salinicola*^TS^** | **MFLUCC 15-1277** | submerged marine wood | Thailand | KX573915 | KX573932 |
| ***Monosporascus cannonballus*^TS^** | **ATCC 26931** | NA | NA | FJ430598 | NA |
| *Monosporascus cannonballus*^TS^ | CMM 3646 | *Boerhavia sp.* | Brazil | JX971617 | NA |
| ***Neoeutypella baoshanensis*^TS^** | **BAP101** | *Pinus armandii* | China | MH822887 | MH822888 |
| *Neoeutypella baoshanensis* ^TS^ | CBS 274.87 | *Ficus carica* | France | AJ302460 | NA |
| *Neoeutypella baoshanensis* ^TS^ | GL08362 | NA | China | JX241652 | NA |
| *Pedumispora rhizophorae*^TS^ | BCC44877 | *Rhizophora apiculata* | Thailand | KJ888853 | NA |
| *Pedumispora rhizophorae*^TS^ | BCC44878 | *Rhizophora apiculata* | Thailand | KJ888854 | NA |
| *Peroneutypa alsophila* | CBS 250.87 | *Arthrocnemum fruticosum* | France | AJ302467 | NA |
| *Peroneutypa curvispora* | HUEFS 136877 | unidentified plant | Brazil | KM396641 | NA |
| ***Peroneutypa diminutispora*** | **HUEFS 192196** | unidentified plant | Brazil | KM396647 | NA |
| ***Peroneutypa indica*** | **NFCCI-4393** | *Suaeda monoica* | India | MN061368 | MN431498 |
| *Peroneutypa kochiana* | F-092373 | *Atriplex halimus* | Spain | AJ302462 | NA |
| ***Peroneutypa longiasca*** | **MFLUCC 17-0371** | *Hevea brasiliensis* | Thailand | MF959502 | NA |
| ***Peroneutypa mackenziei*** | **MFLUCC 16-0072** | undetermined decaying wood | Thailand | KY283083 | NA |
| ***Peroneutypa microasca***  **(*Eutypa microasca*)** | **BAFC 51550** | unidentifed wood | Argentina | KF964566 | KF964572 |
| ***Peroneutypa polysporae*** | **NFCCI-4392** | *Suaeda monoica* | India | MN061367 | MN431497 |
| *Peroneutypa scoparia* | CBS 242.87 | *Robinia pseudoacacia* | France | AJ302465 | NA |
| *Quaternaria quaternata* | GNF13 | *Fagus* sp. | Iran | KR605645 | KY352464 |
| *Quaternaria quaternata* | CBS 278.87 | *Fagus sulvatica* | Switzerland | AJ302469 | NA |
| ***Xylaria hypoxylon*** | **CBS 122620** | NA | Sweden | AM993141 | NA |

Notes: Acronyms: ATCC: American Type Culture Collecton, Virginia, USA; BAFC: Herbarium, Department of Biological Sciences, Faculty of Natural Sciences, Buenos Aires' University, Argentina; CBS: Westerdijk Fungal Biodiversity Institute (CBS-KNAW Fungal Biodiversity Centre), Utrecht, The Netherlands; CFCC: China Forestry Culture Collection Centre, Beijing, China; CMM: Culture Collection of Phytopathogenic Fungi “Prof. Maria Menezes”; CPC: Culture collection of Pedro Crous, The Netherlands; HUEFS: Herbarium of the State University of Feira de Santana; HVFIG: Hoosic Valley Family Interest Group; IRAN…C: Iranian Fungal Culture Collection, Iranian Research Institute of Plant Protection, Tehran, Iran; IPV: Instituto di Pathologia Vegetale, Milan, Italy, and E. and J. Gallo, Modesto, CA; MFLU: Mae Fah Luang University herbarium, Thailand; MFLUCC: Mae Fah Luang University Culture Collection, Thailand; NFCCI: National Fungal Culture Collection of India, India; STEU: Department of Plant Pathology, University of Stellenbosch, South Africa; UCD: University of California, Davis; UFMGCB: Culture Collection of Microorganisms and Cells of the Universidade Federal of Minas Gerais; NA: not applicable. All the new isolates used in this study are marked by an asterisk (*). Ex-type strains are in bold, type species are denoted with the superscript “TS” and the disputable type species are denoted with the superscript “TSQ”.
