# Supplementary material for: The Hidden Diversity of Diatrypaceous Fungi in China
Source: Front Microbiol. 2021 May 31;12:646262. doi: 10.3389/fmicb.2021.646262 (PMC8200573; doi:10.3389/fmicb.2021.646262)
Supplement: Supplementary Table 3 — Synopsis of species of Allocryptovalsa. [file Table_3.DOCX]

**Table S3.** Synopsis of species of *Allocryptovalsa.*

| **Species** | **Host** | **Asci length**  **(μm)** | **Asci width**  **(μm)** | **Ascospores length**  **(μm)** | **Ascospore width**  **(μm)** | **Reference** |
| --- | --- | --- | --- | --- | --- | --- |
| *A. castanea* | *Castanea mollissima* | (52–)60–83(−92) | (11–)12–17(−25) | 8–11(−13) | 2.5–3.5(−4) | This study |
|  | *Juglans regia* |  |  |  |  |  |
| *A. castaneicola* | *Castanea mollissima* | 194–202 | 15–21 | 22–25 | 5–6 | This study |
| *A. cryptovalsoidea* | *Ficus carica* | 65–120 | 15–20 | 8–12(–13.5) | 2–3 | Trouillas et al., 2011 |
| *A. elaeidis* | *Elaeis guineensis* | (55–)68–147(−157) | 14–26 | (6–)7.5–9(−10.5) | 2–4 | Konta et al., 2020 |
| *A. polyspora* | *Hevea brasiliensis* | (33–)70–120(−177) | (4–)13–19(−21) | 5–14 | 2–4 | Senwanna et al., 2017 |
| *A. rabenhorstii* | *Vitis vinifera*  *Sambuscus nigra* | (55−)70−90(−95) | (15−)18−22(−27) | (10−)13.5−15(−17.5) | (3−)4−5(−6) | Trouillas et al., 2011 |
| *A. truncata* | NA | (84–)89–117(–122) | (10–)12–15(–16.5) | (7–)7.2–10.7 | 2.1–2.7 | Hyde et al., 2020b |
