# Supplementary material for: The Hidden Diversity of Diatrypaceous Fungi in China
Source: Front Microbiol. 2021 May 31;12:646262. doi: 10.3389/fmicb.2021.646262 (PMC8200573; doi:10.3389/fmicb.2021.646262)
Supplement: Supplementary Table 4 — Synopsis of species of Diatrypella from Betula spp. [file Table_4.DOCX]

**Table S4.** Synopsis of species of *Diatrypella*.

| **Species** | **Host** | **Asci length**  **(μm)** | **Asci width**  **(μm)** | **Ascospores length**  **(μm)** | **Ascospores width**  **(μm)** | **Reference** |
| --- | --- | --- | --- | --- | --- | --- |
| *Da. betulae* | *Betula albosinensis* | 132–140 | 7.5–10.5(−11.5) | (4.5−)5–7 | 1–2 | This study |
| *Da. betulicola* | *Betula davurica*, *Betula platyphylla* | 117–133 | 10–12 | 5–8 | 1–2 | This study |
| *Da. favacea* | *Betula platyphylla* | 64–124 | (9–)9.5–12(−12.5) | (3.5–)4–5.5(–6) | 1.5–2 | This study |
| *Da. hubeiensis* | *Betula davurica* | 189–240 | 18–21 | 6–8.5(–9) | 1–2 | This study |
| *Da. pulvinata* | NA | 55–77 | 6.6–9 | 6–8 | 1–1.5 | Barr, 1984 |
| *Da. shennongensis* | *Betula albosinensis* | 129–140 | (5–)8–12 | (4.5–)5–6.5(–7) | 1–1.5 | This study |
| *Da. yunnanensis* | NA | 105–210 | 15–30 | 18–22 | 3–4 | Hyde et al., 2020b |
