# Supplementary material for: The Hidden Diversity of Diatrypaceous Fungi in China
Source: Front Microbiol. 2021 May 31;12:646262. doi: 10.3389/fmicb.2021.646262 (PMC8200573; doi:10.3389/fmicb.2021.646262)
Supplement: Supplementary Table 5 — Distribution of diatrypaceous fungi on plants in China. [file Table_5.DOCX]

**Table S5.** Distribution of diatrypaceous fungi on plants in China.

| **Genera** | **Species** | **Host** | **Collection site (Province)** | **Sequence data** | **References** |
| --- | --- | --- | --- | --- | --- |
| *Allocryptovalsa* | *Allocryptovalsa castanea* | *Castanea mollissima, Juglans regia* | Hebei, Yunnan | ITS, tub2 | This study |
| *Anthostoma* | *Anthostoma longiascum* | NA | Anhui | NA | Tai, 1979 |
| *Anthostoma* | *Anthostoma turgidum* | NA | Fujian, Guandong | NA | Tai, 1979 |
| *Cryptosphaeria* | *Cryptosphaeria exornata* | Fraxinus sp. | Heilongjiang, Jilin | NA | Vasilyeva and Ma, 2014 |
| *Cryptosphaeria* | *Cryptosphaeria ligniota* | *Populus tremula* | Heilongjiang, Jilin | NA | Vasilyeva and Ma, 2014 |
| *Cryptosphaeria* | *Cryptosphaeria nigrescens* | *Populus davidiana* | Jilin | NA | Vasilyeva and Ma, 2014 |
| *Cryptosphaeria* | *Cryptosphaeria populina* | *Populus berolinensis* | NA | NA | Tai, 1979 |
| *Cryptosphaeria* | *Cryptosphaeria pullmanensis* | Populus alba, Salix alba, Salix matsudana | Xinjiang | ITS, tub2 | Ma et al., 2016 |
| *Cryptosphaeria* | *Cryptosphaeria venusta* | Betula sp. | Jilin | NA | Vasilyeva and Ma, 2014 |
| *Cryptovalsa* | *Cryptovalsa mangrovei* | Mangrove | HongKong | NA | Inderbitzin et al., 1999 |
| *Cryptovalsa* | *Cryptovalsa protracta* | *Ligustrum lucidum* | Jiangsu | NA | Tai, 1979 |
| *Diatrype* | *Diatrype acericola* | *Acer mandshuricum* | Jilin | NA | Vasilyeva and Ma, 2014 |
| *Diatrype* | *Diatrype albopruinosa* | Maackia amurensis, Padus avium | Heilongjiang | NA | Vasilyeva and Ma, 2014 |
| *Diatrype* | *Diatrype betulae* | *Betula davurica* | Beijing | ITS, tub2 | This study |
| *Diatrype* | *Diatrype bullata* | Salix sp. | Heilongjiang, Hebei, Gansu, Qinghai | NA | Kobayashi and Zhao, 1989;  Tai, 1979 |
| *Diatrype* | *Diatrype castaneicola* | *Castanea mollissima* | Hebei | ITS, tub2 | This study |
| *Diatrype* | *Diatrype chlorosarca* | Rubus sp. | Fujian, Guandong, Guangxi, Guizhou, Yunnan | NA | Tai, 1979 |
| *Diatrype* | *Diatrype disciformis* | NA | Hebei, Jiangsu, Anhui, Hunan, Guangxi | ITS | Tai, 1979 |
| *Diatrype* | *Diatrype enteroxantha* | NA | Yunnan | ITS, tub2 | Tai, 1979 |
| *Diatrype* | *Diatrype hypoxyloides* | *Quercus mongolica, Quercus* sp. | Heilongjiang, Jilin | NA | Vasilyeva and Ma, 2014 |
| *Diatrype* | *Diatrype macounii* | Salix sp. | Heilongjiang | NA | Vasilyeva and Ma, 2014 |
| *Diatrype* | *Diatrype microstroma* | NA | Shanxi, Zhejiang, Guangxi | NA | Tai, 1979 |
| *Diatrype* | *Diatrype platystoma* | Betula sp., Carpinus cordata, Malus sp. | Heilongjiang, Jilin, Hebei, Shanxi, Zhejiang, Anhui,  Hunan, Guangdong, Sichuan, Yunnan | NA | Vasilyeva and Ma, 2014; Tai, 1979 |
| *Diatrype* | *Diatrype princeps* | NA | Gansu, Guangdong | NA | Tai, 1979 |
| *Diatrype* | *Diatrype quercicola* | *Quercus mongolica* | Beijing | ITS, tub2 | This study |
| *Diatrype* | *Diatrype quercina* | Quercus sp. | Hebei, Jiangsu | ITS | Tai, 1979 |
| *Diatrype* | *Diatrype stigma* | *Quercus mongolica* | Heilongjiang, Jilin, Hebei, Shanxi, Gansu, Qinghai, Jiangsu, Zhejiang, Anhui, Hunan, Guangdong, Guangxi, Sichuan, Yunnan | ITS, tub2 | Vasilyeva and Ma, 2014; Tai, 1979 |
| *Diatrype* | Diatrype stigma var. japonica | NA | Hebei, Qinghai, Hunan, Guangxi | NA | Tai, 1979 |
| *Diatrype* | *Diatrype subundulata* | *Acer mandshuricum* | Jilin | NA | Vasilyeva and Ma, 2014 |
| *Diatrype* | *Diatrype undulata* | *Betula sp.* | Heilongjiang, Jilin | ITS | Vasilyeva and Ma, 2014 |
| *Diatrypella* | *Diatrypella deusta* | Palma | Zhejiang, Guangxi | NA | Tai, 1979 |
| *Diatrypella* | *Diatrypella betulae* | *Betula albosinensis* | Hubei | ITS, tub2 | This study |
| *Diatrypella* | *Diatrypella betulicola* | *Betula davurica*, *Betula platyphylla* | Beijing | ITS, tub2 | This study |
| *Diatrypella* | *Diatrypella favacea* | *Betula platyphylla*, Betula sp. | Sichuan, Xinjiang | ITS, tub2 | Tai, 1979; This study |
| *Diatrypella* | *Diatrypella hubeiensis* | *Betula davurica* | Hubei | ITS, tub2 | This study |
| *Diatrypella* | *Diatrypella shennongensis* | *Betula albosinensis* | Hubei | ITS, tub2 | This study |
| *Diatrypella* | *Diatrypella verruciformis* | NA | Hebei, Jilin, Gansu, Qinghai, Jiangsu, Zhejiang, Hunan,  Guangxi | ITS, tub2 | Tai, 1979 |
| *Diatrypella* | *Diatrypella vitis* | Vitis sp. | Zhejiang | NA | Tai, 1979 |
| *Diatrypella* | *Diatrypella yunnanensis* | NA | Yunnan | ITS, tub2 | Hyde et al., 2020 |
| *Endoxylina* | *Endoxylina citricola* | NA | NA | NA | Teng, 1938 |
| *Endoxylina* | *Endoxylina mori* | NA | Taiwan | NA | Sawada, 1959 |
| *Eutypa* | *Eutypa acharii* | NA | Guangdong, Yunnan | NA | Tai, 1979 |
| *Eutypa* | *Eutypa bambusina* | *Gramineae* | Jiangsu, Zhejiang, Anhui, Fujian, Hunan, Guangdong, Guangxi, Guizhou, Yunnan | NA | Tai, 1979 |
| *Eutypa* | *Eutypa heteracantha* | NA | Jilin, Jiangsu | NA | Tai, 1979 |
| *Eutypa* | *Eutypa kusanoi* | NA | Taiwan | NA | Tai, 1979 |
| *Eutypa* | *Eutypa leioplaca* | NA | Guangdong | NA | Tai, 1979 |
| *Eutypa* | *Eutypa ludibunda* | NA | Jiangsu | NA | Tai, 1979 |
| *Eutypa* | *Eutypa milliaria* | NA | Jiangsu, Zhejiang, Guangxi | NA | Tai, 1979 |
| *Eutypa* | *Eutypa spinosa* | NA | Guangdong | ITS | Tai, 1979 |
| *Eutypella* | *Eutypella bambusina* | Bambusa sp. | Guangdong | NA | Tai, 1979 |
| *Eutypella* | *Eutypella capillata* | NA | Jiangsu | NA | Tai, 1979 |
| *Eutypella* | *Allocryptovalsa castaneicola* | *Castanea mollissima* | Hebei | ITS, tub2 | This study |
| *Eutypella* | *Eutypella citricola* | *Citus grandis*, *Morus alba* | Guangxi, Jiangsu | ITS, tub2 | Tai, 1979; This study |
| *Eutypella* | *Eutypella deusta* | NA | Hebei, Jiangsu, Zhejiang, Gunagdong, Gaungxi, Yunnan, | NA | Tai, 1979 |
| *Eutypella* | *Eutypella halseyana* | Betula sp. | Hebei, Jilin, Heilongjiang | NA | Tai, 1979 |
| *Eutypella* | *Eutypella paliuri* | *Paliurus ramosissimus* | Guangxi | NA | Tai, 1979 |
| *Eutypella* | *Eutypella rosae* | Rosa sp. | Hunan, Zhejiang | NA | Tai, 1979 |
| *Eutypella* | *Eutypella sabalina* | *Chanaerops humilis* | Guangdong | NA | Tai, 1979 |
| *Eutypella* | *Eutypella sepulta* | NA | Jiangsu | NA | Tai, 1979 |
| *Eutypella* | *Eutypella stellulata* | NA | Hebei, Jiangsu, Zhejiang, Anhui, Fujian, Hunan, Guangdong, Guangxi, Yunnan | NA | Tai, 1979 |
| *Neoeutypella* | *Neoeutypella baoshanensis* | *Pinus armandii* | Yunnan | ITS, tub2 | Rungtiwa et al., 2019 |
| *Peroneutypa* | Peroneutypa scoparia | NA | Hunan, Jiangsu, Guangdong | ITS | Tai, 1979 |
